# HOMA Index Establishes Similarity to a Reference Molecule

**DOI:** 10.1021/acs.jcim.3c01551

**Published:** 2023-12-06

**Authors:** Jan Cz. Dobrowolski, Sławomir Ostrowski

**Affiliations:** Institute of Nuclear Chemistry and Technology, 16 Dorodna Street, 03-195 Warsaw, Poland

## Abstract

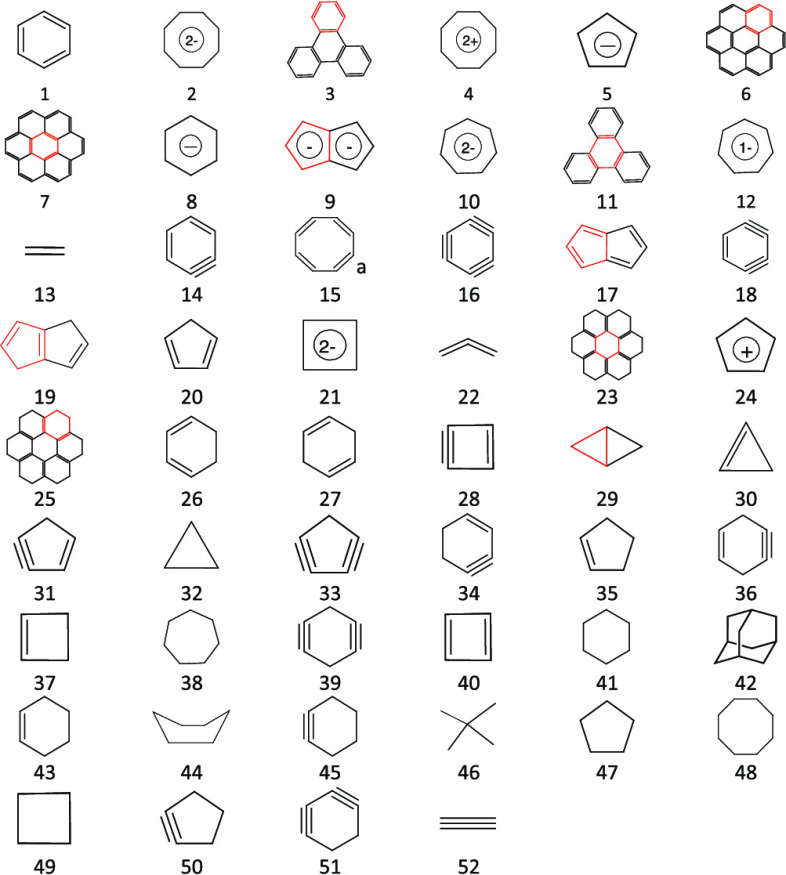

The article shows that the definition of the HOMA index of geometrical aromaticity satisfies the axioms of a similarity function between the examined and benzene ring. Consequently, for purely mathematical reasons, the index works exceptionally well as an index of aromaticity: it expresses a geometric similarity to the archetypal aromatic benzene. Thus, if the molecule is geometrically similar to benzene, then it is also chemically similar, and therefore, it is aromatic. However, the similarity property legitimizes using the HOMA-like indices to express similarity to molecules other than benzene, whether cyclic or linear and existing or hypothetical. The paper demonstrates an example of HOMA-similarity to cyclohexane, which expresses a (relaxed)-saturicity property not accompanied by strong structural strains or steric hindrances. Further, it is also shown that the HOMA index can evaluate the properties of whole molecules, such as 25 unbranched catacondensed isomers of hexacene. The index exhibits a significant quadratic correlation with the total energy differences of planar isomers from which the nonplanar ones deviate. Moreover, the HOMA index of hexacene isomers significantly correlates with the Kekulé count connected to the resonance energy in the Hückel approximation. As a result, the study shows that the HOMA index can be used not only for aromaticity analyses but also as a general chemical descriptor applicable to rings, chains, composed molecular moieties, or even whole molecules.

## 1 Introduction

Aromaticity is not an observable molecular property, although it is essential in modern chemistry. It manifests itself in multiple molecular characteristics and is thus defined in various ways, highlighting its unique features. Different definitions expose the specifics of molecular geometry, reactivity, additional energy stabilization, magnetic currents, and some other features and sometimes disagree. Therefore, it is imperative to understand every aspect of the aromaticity definition. This study is focused on analyzing the definition of the Harmonic Oscillator Model of Aromaticity, HOMA, and geometrical aromaticity index.^1^ It is one of the most successful and widely used measures of aromaticity^2−4^ despite its foundation being grounded on a simple approximative quantum chemical model. Nevertheless, at the very outset, the authors stated that the index “*is quite independent/in principle/of quantum chemical models*”.^1^ Hereafter, we demonstrate why it is the absolutely correct statement.

For hydrocarbon rings, HOMA is defined as follows
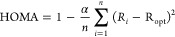
1where *R*_i_ and *R*_opt_ (Å) stand for the *i*-th CC bond length in the analyzed ring and the reference benzene ring (*R*_opt_ = *R*_B_) for which HOMA = 1, respectively, *n* is number of CC bonds in the ring, and α = 257.7 Å^–2^ normalizes the index to be unitless and equal to 0 for a hypothetical perfectly alternating Kekulé cyclohexatriene ring.

HOMA can be reformulated in terms of two destabilizing factors: energetic (EN) and geometric (GEO), disrupting the perfect aromaticity, for which HOMA = 1.^5−7^
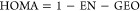
2
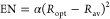
3
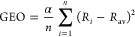
4where *R*_av_ is the averaged bond length in the examined ring, EN is a normalized squared difference of the bond length arithmetic means, and GEO is a normalized bond variance in the examined molecule.

First defined for aromatic hydrocarbons, HOMA was then parametrized in several ways to evaluate the geometrical aromaticity of heterocyclic structures:^3,7−11^ The HOMA index has also been defined as a function of ring bond properties other than bond lengths (*R*) like parameters of the electron density in bond critical points.^12−14^ Interestingly, the HOMA index asymptotically converges as the number *n* of molecular units increases. For example, for alkanes, spiro cyclopropanes, polyynes, and allenes, the index decreases with the increasing *n* and converges to negative values from −4 to −2.^13^ This can suggest an increase in the degree of insulation with an increase in the number of units. For alkenes and polythiophenes, the index increases and converges to values above 0.6, while for polypyrrole, it reaches ca. 0.8.^13,15^ This can suggest the increase of π-electron delocalization with an increase in the number of units.^13,16^ The HOMA indices were also used to study linear and branched alkanes, and for *n*-alkanes, a good correlation with the boiling points was found. On the other hand, for *n*-alkane constitutional isomers, the HOMA index tends to increase with the boiling points from the most branched to the most extended isomer.^17^

The above data indicate that the HOMA index is more than just a geometrical aromaticity index. However, why it may be so is known.^16^ First, the HOMA index is connected to a distance in the abstract *n*-dimensional molecular space and, second, to a statistical value such as a variance.^17^ Third, the association of HOMA with aromaticity is only through *R*_opt_, taken from the aromaticity standard: benzene. The α factor establishes HOMA = 0 for a nonaromatic, hypothetical cyclohexatriene molecule.

This study shows that HOMA expresses a similarity between the studied and reference moiety. If a molecule is geometrically similar to benzene, HOMA-similar, then it also is chemically similar and, thus, aromatic. By replacing benzene with a different reference molecule, the HOMA index with this new reference provides geometrical similarity to the new reference. Consequently, the similarity of the chemical properties of the new reference is also satisfied. Thus, the HOMA index referred to benzene measures aromaticity, but when referred to cyclohexane, it provides similarity to an unstrained, saturated hydrocarbon. We demonstrated that HOMA can also be used to evaluate the properties of entire molecules, such as isomers of hexacene. Ultimately, we argue that the HOMA index can describe a general geometric similarity between chemical moieties and can be shaped according to the unconstrained will of a researcher rather than the form intended by its inventors.

## 2 Computations

All structure optimizations were done with the DFT calculations using the B3LYP functional,^18,19^ along with the D3 Grimme correction for dispersion forces,^20^ the 6-31G** Pople-type basis set,^21^ and Gaussian 09 software.^22^ The 6-31G** basis set was chosen to enable the reproduction of the calculations in any laboratory and because this small basis set yields sufficiently good geometry, energy, and electron density data^23^ to build organic physical chemistry indices. It was confirmed that each structure exhibits proper symmetry and is at the energy minimum, owning all positive harmonic frequencies. The XYZ coordinates as well as the original input and output Gaussian 16 files of all molecules calculated are given in the Supporting Information.

## 3 Results and Discussion

### 3.1 EN and GEO Factors

Let us denote by *x*_1_,*x*_2_,···,*x*_*n*_ and *y*_1_,*y*_2_,···,*y*_*n*_ the CC bonds in the analyzed and reference rings *x* and *y*, respectively. Then, EN factor (3) can be redrawn as the square of the difference between the CC bond lengths arithmetic means *x̅* = *R*_av_ and *y̅* = *R*_opt_ normalized by α
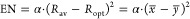
5

The GEO factor (4) is then a normalized variance of the CC bond lengths in the examined moiety *x*
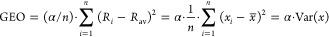
6

Thus
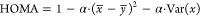
7

So, to calculate the HOMA index, it is enough to know the appropriate arithmetical means and the variation of the CC bond lengths in the single moiety *x*. Note that only the GEO = α·Var(x) term contains information about the ring size hidden in the summation operation. For the rings other than six-membered, the summation stops at *n* ≠ 6, but still, *R*_opt_ = *R*_B_ is used. So, it is tacitly assumed that in every reference ring, the CC distance equals that in benzene, even though the matching *n*-membered hydrocarbon ring does not exist. Hence, HOMA = 1 for every reference ring consisting of *k* = 3, 4, 5, 7,··· bonds, and it is as ideally aromatic as benzene. However, if arithmetical means of comparing moieties and variation of the ring bond lengths in one of them are sufficient to determine HOMA, then even a single distance can play a role in the reference moiety. It can be present in a selected molecule but can also be equal to the arithmetic mean of the bond lengths in an existing or a hypothetical moiety.

### 3.2 HOMA Establishes a Similarity between Moieties

A similarity between two elements *x* and *y* of a set *X* is a function  which for any *x*,*y* ∈ *X* satisfies the following axioms^24^
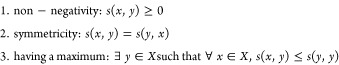
8

Notice that if α = 1, then HOMA becomes a similarity function *s*_HOMA_(x,y)
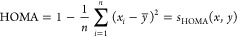
9

The non-negativity is satisfied since for hydrocarbons 0 < (*x*_*i*_ – *y̅*)^2^ < 1, so ; thus, . The symmetricity is given by the symmetricity of the (*x*_*i*_ – *y̅*)^2^ factors. Eventually, if *x*_*i*_ = *y̅* for any *i*, then HOMA = *s*_HOMA_ (*y*,*y*) = 1 is the maximum. Notice that HOMA truncated to a single EN factor, 1 – EN, for α = 1 is the similarity function for analogous reasons. Notably, HOMA is connected also to a distance in abstract molecular space.^17^

However, the HOMA non-negativity is not essential in studying the aromaticity of molecules. Notice that α ≈ 250 > 1 provides most figures between 1 and ca. −10, which have been convenient so far. Besides, returning to the strict similarity function is trivial. Thus, for chemical purposes, let us use a similarity-like function satisfying only symmetricity and having maximum axioms (8). Then, the HOMA and HOMA shortened to the single EN factor functions are just such “chemical” similarity functions.

Therefore, for the hexahydroxy and hexafluoro benzene, C_6_(OH)_6_ and C_6_F_6_, the rings are geometrically nearly identical to that of benzene, HOMA ≈ 1.^17^ The side ring in triphenylene is quite similar to benzene (HOMA = 0.93, Table 1). The off-center coronene and C_6_Li_6_ rings are less similar to benzene. Indeed, the CC bonds in the former vary, and though the latter has *D*_6h_ symmetry, the CC bonds are longer than those in benzene. Hence, they have HOMA ≈ 0.8,^17^ meaning smaller aromaticity. HOMA of the ring in cycloheptatriene or pentalene dianions, C_7_H_7_^2–^ and C_8_H_6_^2–^, equals ca. 0.5 (Table 1). Although nothing seems similar between the five-membered pentalene dianion and benzene rings, in terms of similarity established by the HOMA function, they are somehow aromatic, which for C_8_H_6_^2^ is confirmed by the NICS index.^25^ Again, according to (7), HOMA-similarity requires similar CC bond arithmetic means in the studied and reference rings and slight variation of the CC bonds in the former. It is difficult to judge whether the central ring in triphenylene (HOMA = 0.28) is still similar to benzene or not. Unsurprisingly, ethene and cyclooctatetraene are HOMA-dissimilar to benzene and are not aromatic: HOMA ≈ 0.1 and −0.1, respectively (Table 1). The CC bond in the former is double and radically different from that in aromatic benzene, whereas the double and single CC bonds in the latter alter, and the molecule has a significant GEO factor. In agreement with the intention of the HOMA index founders,^1^ similarity with benzene ends at HOMA = 0, reflecting the nonaromaticity of the perfectly alternating Kekulé cyclohexatriene ring. However, according to HOMA, chair cyclohexane and archetypically antiaromatic cyclobutadiene rings have similar HOMA indices around −4.4 (Table 1). Moreover, the HOMA of the ring in antiaromatic pentalene is close to 0. Thus, the HOMA-similarity with benzene does not allow for distinguishing between cycloalkane, nonaromatic, and antiaromatic hydrocarbons. We shall check whether such differentiation could be possible by going beyond the primary definition in Section 3.3.2.

**Table 1:** HOMA_B_ and HOMA_c_ Indices Calculated Using, Respectively, Benzene (B) and Chair Cyclohexane (C) Reference Molecules^a^

molecule/moiety	#	HOMA_B_	HOMA_C_	molecule/moiety	#	HOMA_C_	HOMA_B_
benzene	1	1.00	–4.53	chair cyclohexane	41	1.00	–4.53
cyclooctatetraene dianion	2	0.94	–3.46	adamantane	42	1.00	–4.58
side ring in triphenylene	3	0.93	–4.14	neopentane	46	1.00	–4.74
cyclooctatetraene dication	4	0.89	–3.07	cycloheptane	38	1.00	–4.32
cyclopentadienyl anion	5	0.85	–2.88	boat cyclohexane	44	0.99	–4.61
side ring in coronene	6	0.81	–3.30	cyclooctane	48	0.99	–4.90
central ring in coronene	7	0.75	–2.43	cyclopentane	47	0.98	–4.81
benzene anion radical	8	0.61	–3.15	cyclobutane	49	0.97	–5.39
ring in pentalene dianion	9	0.57	–2.00	cyclopropane	32	0.86	–2.90
cycloheptatriene dianion	10	0.52	–2.37	ring in bicyclo[1.1.0]butane	29	0.36	–2.21
central ring in triphenylene	11	0.28	–1.65	central ring in 12*H*-coronene	23	0.20	–1.13
cycloheptatriene anion	12	0.17	–3.76	cyclobutadiene dianion	21	0.10	–1.04
ethene	13	0.07	–9.99	cyclohexene	43	–0.91	–4.59
benzyne	14	0.06	–7.03	cyclopentene	35	–1.33	–3.08
cyclooctatetraene	15	–0.15	–4.82	cyclobutene	37	–1.59	–3.79
C_6_ cyclic molecule	16	–0.20	–10.89	central ring in triphenylene	11	–1.65	0.28
ring in pentalene	17	–0.22	–3.32	cyclobuta-1-yn-3-ene	28	–1.70	–1.97
cyclohexa-1,3-diyn-5-ene	18	–0.45	–8.94	ring in pentalene dianion	9	–2.00	0.57
ring in 1,4-dihydropentalene	19	–0.66	–3.01	off-center ring in 12*H*-coronene	25	–2.25	–1.25
cyclopentadiene	20	–0.93	–2.97	cycloheptatriene dianion	10	–2.37	0.52
cyclobutadiene dianion	21	–1.04	0.10	central ring in coronene	7	–2.43	0.75
allene	22	–1.08	–13.39	cyclohexa-1,4-diene	27	–2.72	–1.53
central ring in 12*H*-coronene	23	–1.13	0.20	cyclohexa-1,3-diene	26	–2.75	–1.48
cyclopentadienyl cation	24	–1.16	–3.79	cyclopentadienyl anion	5	–2.88	0.85
off-center ring in 12*H*-coronene	25	–1.25	–2.25	cyclopentadiene	20	–2.97	–0.93
cyclohexa-1,3-diene	26	–1.48	–2.75	ring in 1,4-dihydropentalene	19	–3.01	–0.66
cyclohexa-1,4-diene	27	–1.53	–2.72	cyclooctatetraene dication	4	–3.07	0.89
cyclobuta-1-yn-3-ene	28	–1.97	–1.70	benzene anion radical	8	–3.15	0.61
ring in bicyclo[1.1.0]butane	29	–2.21	0.36	side ring in coronene	6	–3.30	0.81
cyclopropene	30	–2.70	–3.91	ring in pentalene	17	–3.32	–0.22
cyclopenta-1-yn-3-ene	31	–2.78	–5.49	cyclooctatetraene dianion	2	–3.46	0.94
cyclopropane	32	–2.90	0.86	cycloheptatriene anion	12	–3.76	0.17
cyclopentadiyne	33	–2.98	–5.51	cyclopentadienyl cation	24	–3.79	–1.16
cyclohexa-1-yn-3-ene	34	–2.98	–5.47	cyclohexyne	45	–3.90	–4.68
cyclopentene	35	–3.08	–1.33	cyclopropene	30	–3.91	–2.70
cyclohexa-1-yn-4-ene	36	–3.12	–5.34	side ring in triphenylene	3	–4.14	0.93
cyclobutene	37	–3.79	–1.59	cyclobutadiene	40	–4.43	–4.33
cycloheptane	38	–4.32	1.00	cyclopentyne	50	–4.52	–5.66
cyclohexa-1,4-diyne	39	–4.32	–8.02	benzene	1	–4.53	1.00
cyclobutadiene	40	–4.33	–4.43	cyclooctatetraene	15	–4.82	–0.15
chair cyclohexane	41	–4.53	1.00	cyclohexa-1-yn-4-ene	36	–5.34	–3.12
adamantane	42	–4.58	1.00	cyclohexa-1-yn-3-ene	34	–5.47	–2.98
cyclohexene	43	–4.59	–0.91	cyclopenta-1-yn-3-ene	31	–5.49	–2.78
boat cyclohexane	44	–4.61	0.99	cyclopentdiyne	33	–5.51	–2.98
cyclohexyne	45	–4.68	–3.90	benzyne	14	–7.03	0.06
neopentane	46	–4.74	1.00	cyclobutyne	53	–7.43	–10.69
cyclopentane	47	–4.81	0.98	cyclohexa-1,4-diyne	39	–8.02	–4.32
cyclooctane	48	–4.90	0.99	cyclohexa-1,3-diyn-5-ene	18	–8.94	–0.45
cyclobutane	49	–5.39	0.97	ethene	13	–9.99	0.07
cyclopentyne	50	–5.66	–4.52	cyclohexa-1,3-diyne	51	–10.51	–7.75
cyclohexa-1,3-diyne	51	–7.75	–10.51	C_6_ cyclic molecule	16	–10.89	–0.20
ethyne	52	–7.82	–27.33	allene	22	–13.39	–1.08
cyclobutyne	53	–10.69	–7.43	ethyne	52	–27.33	–7.82

a d(CC_B_)=1.3962 Å, d(CC_C_) = 1.5428 Å, α = 257.7 Å^–2^. The calculations were performed at the B3LYP/6-31G** level with a Grimmes D3 empirical dispersion correction. For molecule numbers, see Figure 1. Aromatic rings are intentionally underrepresented.

Finally, let us emphasize that the HOMA-similarity criterion is not always intuitive and should be operated deliberately. Equation 7 states that a molecule must have similar average bond lengths and as slight bond length variation as possible to be similar to the reference one. An unreflective HOMA analysis of the CC distances in tetracyano, tetrathiocyanato, and 1,1-difluoro-2,2-dichloro ethylene, C_2_(CN)_4_, C_2_(NCS)_4_, and F_2_C=CCl_2_, with the CC distances at the B3LYP/D3/6-31G** level equal to 1.3719, 1.3773, and 1.3351 Å, respectively, would lead to the conclusion that the first two molecules are aromatic, while the third is nonaromatic, as HOMA equals 0.85, 0.91, and 0.04, respectively. Therefore, the HOMA analysis must always be placed in a narrowly defined chemical context.

### 3.3 HOMA as a General Chemical Index

#### 3.3.1 Similarity to a Molecule Other Than Benzene

Several hydrocarbons other than benzene have all identical CC bonds and could serve as a reference for the HOMA analysis: chair cyclohexane (*D*_3d_ symmetry), adamantane (*T*_d_), cyclobutane (*D*_2d_), cyclopropane (*D*_3h_), cyclopentadienyl anion (C_5_H_5_^–^, *D*_5h_), cyclooctatetraenyl anion and cation (C_8_H_8_^–^, C_8_H_8_^+^, *D*_5h_), and cycloheptatrienyl cation (C_7_H_7_^+^, *D*_7h_), while the anion has slightly lowered symmetry by the Jahn–Teller effect.^26^ The persubstituted molecules listed above, like, e.g., hexamethylbenzene,^27^ or fragments of a larger symmetric structure, such as the central ring in coronene, also have all identical CC bonds. Nevertheless, one-CC-bond molecules are the limit case eq 7 allows. They would set the HOMA-similarity to the most basic organic molecules such as ethene or acetylene.

Now, let us replace *R*_opt_ = *R*_B_ = 1.3962 Å with *R*_C_ = 1.5428 Å but keep α = 257.7 Å^–1^ (*B* and *C* denote benzene and cyclohexane, respectively). Then, HOMA_C_(C) = 1.0000 and HOMA_C_(B) = −4.5338, and HOMA_C_(B) = HOMA_B_(C) = −4.5. An intuition on what the HOMA_C_-similarity to cyclohexane means can be shaped to some extent based on Table 1. Tricyclic adamantane, acyclic and branched neopentane, and unstrained cycloheptane are exceptionally similar to cyclohexane. Indeed, they are even more HOMA_C_-similar than the boat cyclohexane. The more strained the cyclic alkane (cyclopentane, cyclobutane, cyclopropane), the less HOMA_C_-similar it is. After all, the three-membered ring of bicyclo[1.1.0]butane (HOMA_C_ ≈ 0.36), the central ring in coronene (HOMA_C_ ≈ 0.20), and the cyclobutadiene dianion (HOMA_C_ ≈ 0.10) already seem to be not similar to cyclohexane.

Once a molecule has an unsaturated bond, it becomes utterly dissimilar from cyclohexane (Table 1, Figure 1). The most dissimilar are acetylene and allene, C_6_ ring molecule, cyclohexa-1,3-diyne, and ethene molecules (HOMA_C_ between −27.5 and −10.00). HOMA_C_ of cyclooctatetraene dianion, cyclooctatetraene dication, cyclopentadienyl anion, side ring in coronene, ..., and pentalene anion ring is from ca. −4.5 to −2.3. However, nonaromatic structures such as side rings in 12H-coronene, cyclohexa-1,3-diene, cyclohexa-1,4-diene, cyclopropene, and cyclohexyne also fall into the same HOMA_C_ range.

**Figure 1:**
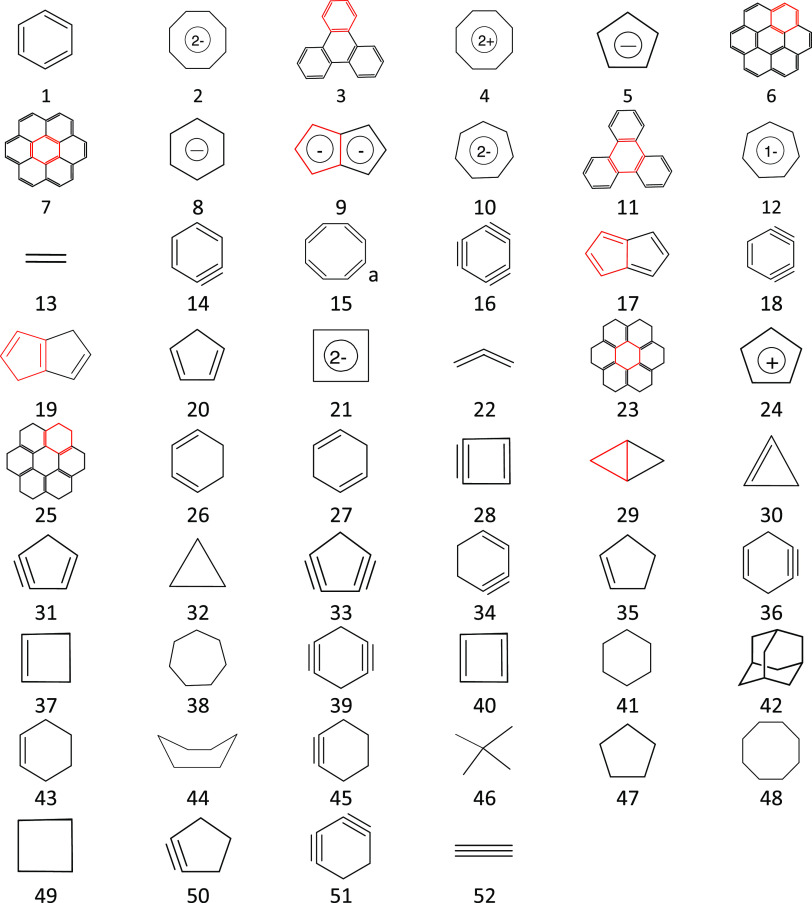
Structures for which the HOMA_B_ and HOMA_C_ indices were calculated (Table 1).

Remark that except for saturated not-strained cyclic adamantane and cycloheptane, HOMA_C_ of neopentane equals 1.00, and HOMA_C_ of isopentane, isohexane, hexane, pentane, and butane exceeds 0.97 (Table 1). Thus, for a moiety to be HOMA_C_-similar, saturation instead of molecular cyclicity is necessary. Such a similarity allows for a moderate strain as in cyclobutane (HOMA_C_ = 0.96) or a much larger one as in cyclopropane (HOMA_C_ = 0.85) or even in cubane (HOMA_C_ = 0.80). Still, a three-membered ring in bicyclo[1.1.0]butane exhibits HOMA_C_ = 0.36, and if the six-membered ring is flat as in 12*H*-coronene, in which each C atom is double-bonded with the external rim, HOMA_C_ = 0.20. The index of very strained, elusive tetrahedrane is equal to −0.81. Thus, the HOMA_C_ index expresses a (*relaxed*)-*saturicity* not accompanied by strong structural strains or steric hindrances.

**Figure 2:**
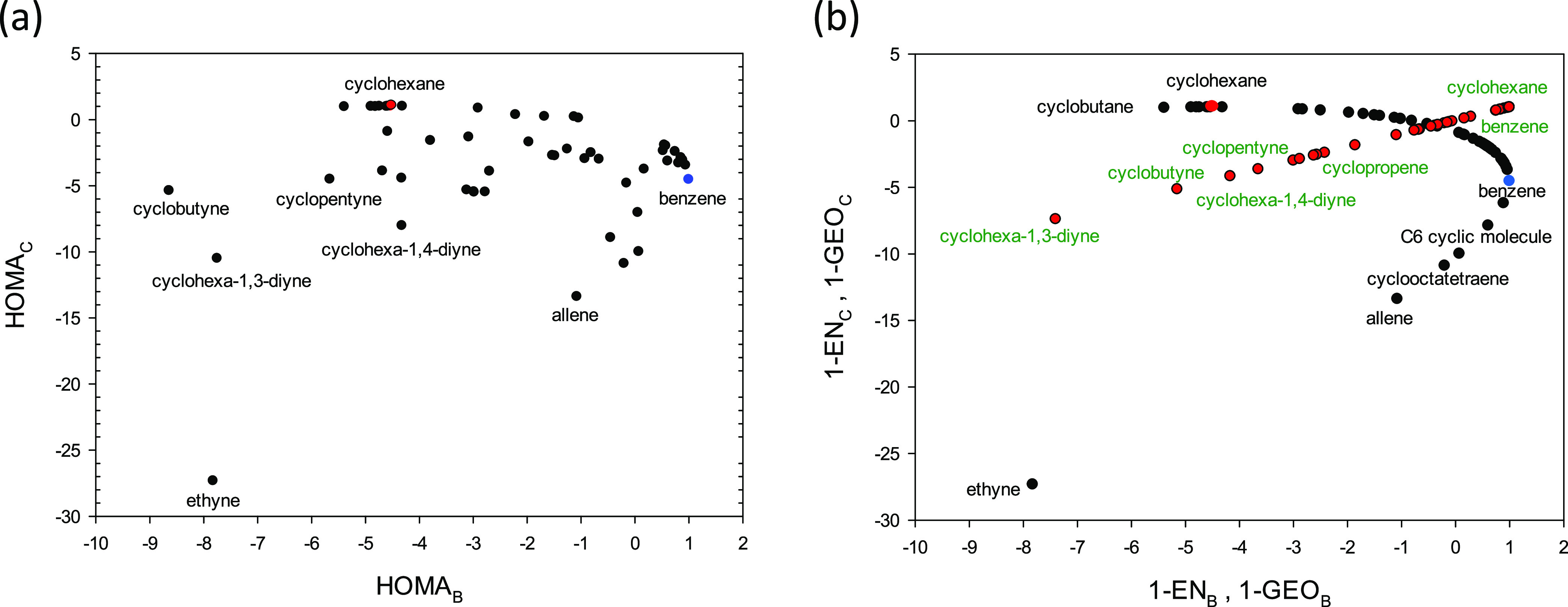
Relationships between indices calculated using benzene (B) and chair cyclohexane (C) reference molecules. The HOMA indices (a) and the HOMA indices truncated to single EN (black points) and GEO factors (red points) (b).

To better see the difference between using the HOMA_B_ and HOMA_C_ indices, let us look at the HOMA_C_(A) = *f*(HOMA_B_(A)) function, where A stands for an arbitrary moiety (Figure 2a). At first glance, the points are spread erratically. Nevertheless, at closer inspection, a regular, nonlinear boundary can be seen (Figure 2a). One boundary branch goes through ethyne, allene, and benzene, and the other through cyclohexane and similar compounds. The former seems composed of primarily unsaturated compounds, while the other is composed of nonstrained structures, irrespectively saturated or not. All interior points seem to correspond to strained and simultaneously unsaturated hydrocarbons.

Notwithstanding partial chaos present in Figure 2a, the HOMA(A) decomposition into the EN(A) and GEO(A) factors reveals where the irregularities come from. The plot of the HOMA(A) function truncated to the 1-EN(A) term forms the border curve where all of the moieties are positioned (black points, Figure 2b). The HOMA(A) function truncated to the 1-GEO(A) term establishes a half straight line (red points, Figure 2b). All of the moieties with GEO = 0 are in the point (1,1) - the beginning of the half straight line. The benzene and cyclohexane molecules and all of the moieties are located at the border curve in Figure 2a. Thus, the border is formed by moieties that have all CC bonds of equal lengths and thus GEO = 0, whereas the interior is formed by the moieties that have (significant) bond length alteration. Seemingly, the curve in Figure 2b is a deformed parabola, but the rotation of this curve by 45° using a pair of parametric equations shows that it is nothing but a parabola (Figure S1 in the Supporting Information).

#### 3.3.2 Similarity to a Molecule with a Nonzero GEO Factor

Now, the feasibility of discrimination between nonaromatic and antiaromatic hydrocarbons is addressed using the HOMA-similarity function. The former compounds should exhibit single and double bond alternation in the ring, as in the elusive cyclohexatriene, while the latter should have 4 instead of 6 π-electrons per ring, as Hückel’s rule for the aromatic systems predicted. The HOMA_B_ index does not allow for distinguishing these species classes (Section 3.2). However, the HOMA_C_ index enables an additional qualification: the nonaromatic and antiaromatic rings have a negative HOMA_C_ index, and they are dissimilar to cyclohexane (Table 1). Hence, the necessary, though insufficient, condition of being a non- or antiaromatic ring is to be HOMA_B_ and HOMA_C_ dissimilar. Immediately a question appears of whether a sufficient condition cannot be formulated using the HOMA index. The answer would be positive if we could find good references for these hydrocarbons.

The first problem in finding such references is the presence of the GEO factor in the archetypical cyclohexatriene and cyclobutadiene rings. In such a case, the condition of the HOMA-similarity should be supplemented by comparing the GEO factors. Hence, expression 7 has to be rewritten in a new HOMA^Δ^ form as follows
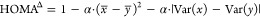
10where module of variances |·| is necessary to conserve the having a maximum axiom (8) for HOMA^Δ^.

To calculate the HOMA_CHT_^Δ^-similarity to cyclohexatriene (CHT), it is necessary first to know the averaged CC distances and their variation in the cyclohexatriene moiety. From Krygowski’s original paper addressing the parameters of the HOMA index for cyclohexatriene,^28^ the following relationship between the bond distances and the parameter α emerges^17^
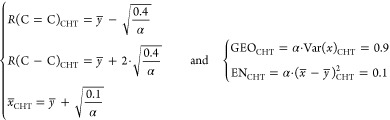
11

In the case of the B3LYP/D3/6-31G** calculations used throughout this study: *y̅* = *R*_opt_ = *R*_B_ = 1.39621 Å and α = 257.7 Å^–2^, *R*(C=C)_CHT_ = 1.35681 Å, *R*(C–C)_CHT_ = 1.47501 Å, and Var(x)_CHT_= 0.00349 Å^2^.

According to HOMA_CHT_^Δ^, the following moieties from Table 1 are the most similar to cyclohexatriene: ring in pentalene, cycloheptatriene anion, cyclooctatetraene, ring in 1,4-dihydropentalene, butadiene, and benzyne: their HOMA_CHT_^Δ^ is equal to 0.91, 0.81, 0.77, 0.57, 0. 54, and 0.52, respectively. This sequence shows that the HOMA_CHT_^Δ^ similarity criterion is not discriminative. Indeed, pentalene, 1,4-dihydropentalene, and the cycloheptatriene anion in the singlet state are antiaromatic for their 4n π electrons per ring^29^ and the NICS index criterion for the anion.^30^ Nonplanar cyclooctatetraene with alternating single and double bonds is nonaromatic. Butadiene has bond length alternation but is not cyclic, and we are not inclined to call it nonaromatic in the sense that the cyclohexatriene and cyclooctatetraene rings are. The structural formula of benzyne (cyclohexa-1-*yn*-3,5-diene) is formally similar to cyclohexatriene’s but, according to the NICS criterion, is more aromatic than benzene.^31^

Let us therefore apply the HOMA_CHT_^Δ^ index to rings very close to cyclohexatriene. A search for rings similar to “Kekulé benzene” among a series of triply fused hexasubstituted benzenes with the *C*_3_ axis using HOMA_B_ = 0 as a criterion demonstrated that only some molecules fulfilling HOMA_B_ ≈ 0 met another necessary condition such that R(C–C) > R_B_ > R(C=C).^17^ However, most of the molecules satisfying the two necessary conditions contained the reactive (antiaromatic) pentalene system fused to the central ring. Such compounds are inappropriate as model reference molecules.

**Figure 3:**
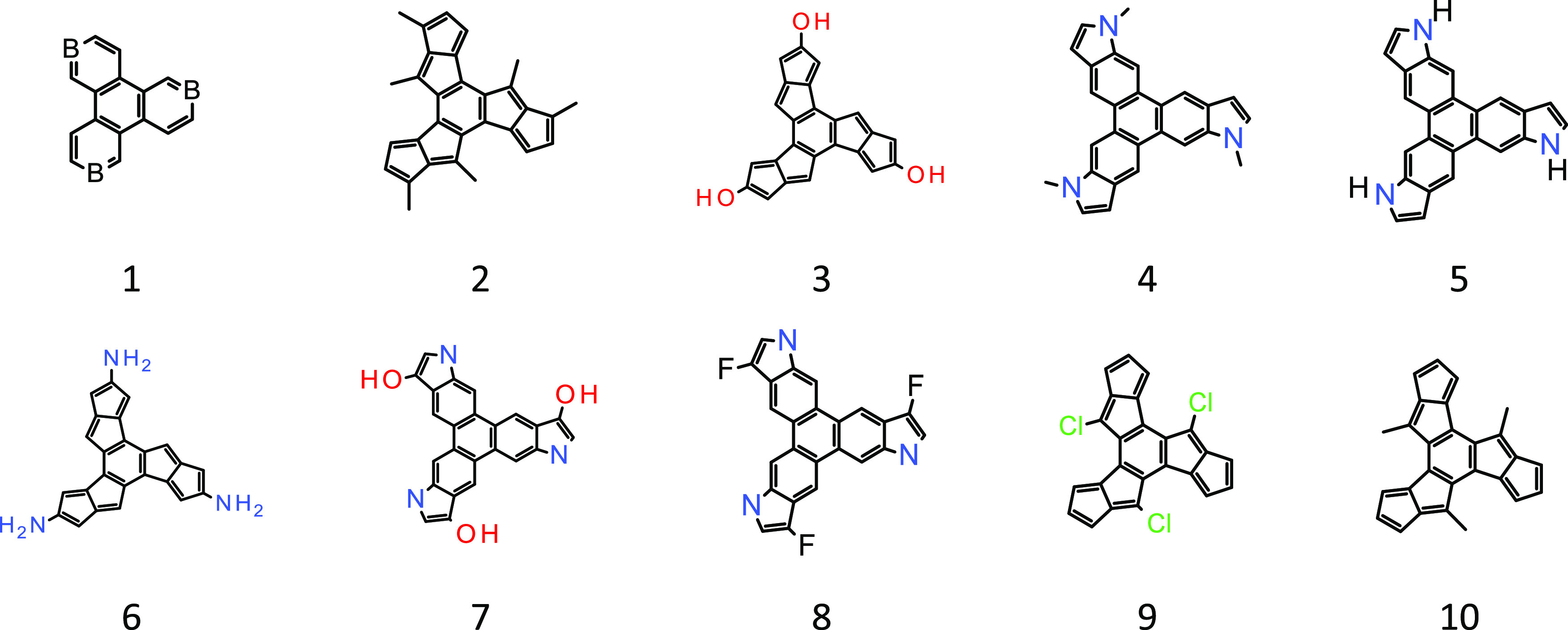
Structures in which the central 6-membered ring has HOMA_B_ ≈ 0, but only 2, 3, 6, 9, and 10 have this ring are similar to cyclohexatriene according to the HOMA_CHT_^Δ^ criterion (see Table 2).

Application of the HOMA_CHT_^Δ^ criterion to the molecules exhibiting HOMA_B_ ≈ 0 considered before^17^ shows that HOMA_CHT_^Δ^ exhibits similarity to cyclohexatriene only for those molecules that satisfied R(C–C) > R_B_ > R(C=C), whereas for the others, HOMA_CHT_^Δ^ is close to 0 or negative (Table 2). This demonstrates that eq 10 can help search for moieties similar to rings with significant GEO factors and that the search can be more effective than using the classical HOMA index supplemented by additional criteria (Figure 3).^17^

**Table 2:** HOMA_B_ and HOMA_CHT_^Δ^ Indices Calculated for the Molecules Shown in Figure 3, Using, Respectively, Benzene (B) and Cyclohexatriene (CHT) Reference Molecules (See Table 1 and Eqs 10 and 11, Respectively)^a^

#	HOMA_B_	EN_B_	GEO_B_	HOMA_CHT_^Δ^	EN_CHT_	GEO_CHT_	mean CC	variance
1	0.06	0.87	0.07	–0.21	0.38	0.83	1.4542	0.00028
**2**	**0.04**	**0.26**	**0.71**	**0.77**	**0.04**	**0.19**	**1.4279**	**0.00274**
**3**	**0.03**	**0.18**	**0.80**	**0.88**	**0.01**	**0.10**	**1.4226**	**0.00309**
4	0.02	0.89	0.10	–0.19	0.39	0.80	1.4549	0.00037
5	0.01	0.89	0.10	–0.20	0.40	0.80	1.4551	0.00038
**6**	**0.00**	**0.20**	**0.80**	**0.89**	**0.02**	**0.10**	**1.4238**	**0.00311**
7	–0.01	0.92	0.09	–0.22	0.41	0.81	1.4558	0.00035
8	–0.01	0.92	0.09	–0.22	0.41	0.81	1.4559	0.00035
**9**	**–0.04**	**0.27**	**0.77**	**0.83**	**0.04**	**0.13**	**1.4285**	**0.00299**
**10**	**–0.05**	**0.26**	**0.78**	**0.84**	**0.04**	**0.12**	**1.4283**	**0.00303**

a The calculations were performed at the B3LYP/D3/6-31G** level with the Grimmes *D*3 empirical dispersion correction. Molecule numbering is as in Figure 3. Moieties 2, 3, 6, 9, and 10 are similar to cyclohexatriene shown in bold.

A moderate success of the HOMA_CHT_^Δ^ index in examining the similarity to cyclohexatriene prompts the supposition that a good index can also be constructed for antiaromatic compounds. However, the issue of a good reference system is returned. The strain of the ring of the antiaromatic cyclobutadiene determines its HOMA_CBD_^Δ^ index. Therefore, it is inadequate as a reference for antiaromaticity. On the other hand, the similarity of less strained antiaromatic pentalene to cyclohexatriene makes discrimination between nonaromatic and antiaromatic hydrocarbons using sole HOMA^Δ^ similarity function doubtful. The sole representation of the 4*n* π-electron requirement in the bond lengths and their variations may not be sufficiently specific. However, further study of this issue goes beyond this project.

At the end of this section, let us mention that the HOMA^Δ^ index can also be constructed based on a reference free of any symmetry constraints. If one assumes the averaged bond length *y̅* and Var(*y*) in eq 10 are taken from an arbitrary asymmetric reference, then the HOMA^Δ^ value shows the HOMA^Δ^-similarity to such a symmetryless compound. This can help assess the similarity to asymmetric compounds such as isooctane, on which the octane number is based, but perhaps also to important natural products such as terpenes. Although, for an asymmetric reference, the analysis could become fuzzier, without further detailed research, it is impossible to assess the benefits of such an approach. However, detailed research on this issue again goes beyond this project.

#### 3.3.3 HOMA as an Index of the Entire Molecule

**Figure 4:**
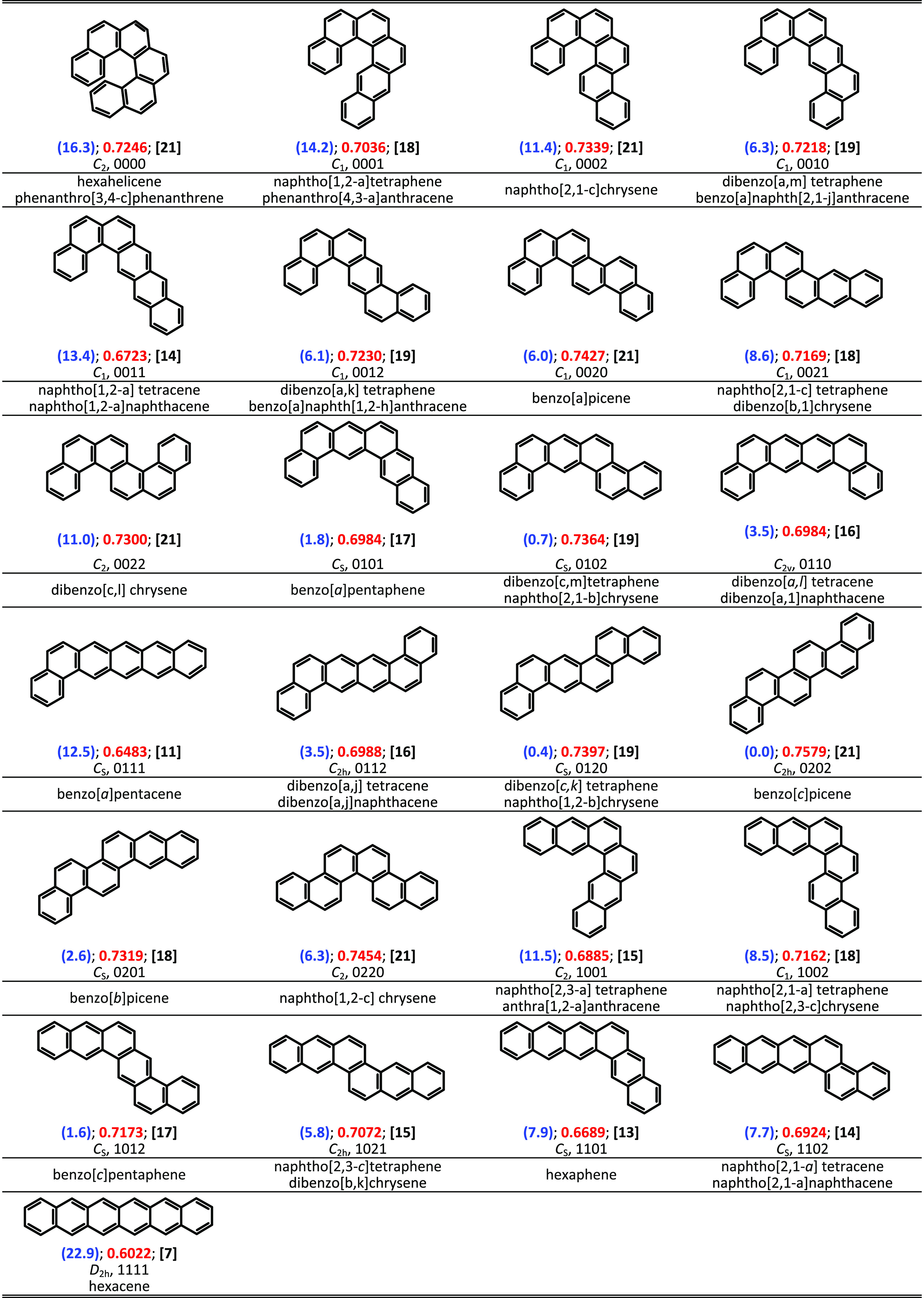
Structural formulas of all unbranched catacondensed hexacene isomers. Total energy differences vs benzo[c]picene are in blue and parentheses (kcal/mol), HOMA_B_ of the entire structure are in red, and the Kekulé count are in black and brackets. Point group symmetry and the PAH 3-digit code^34^ are in the second row in black.

Consider all 25 unbranched catacondensed isomers of hexacene.^32^ Let us calculate the HOMA_B_ index of the entire structures rather than of the individual rings (Figure 4). This can be done using expressions 1 or 7, in which now *n* denotes the number of all CC bonds in the structure instead of only in the single ring. The HOMA_B_ values range from 0.76 for benzo[c]picene to 0.60 for hexacene (Figure 4). Notably, the former is the most stable, and the latter is the least stable. Also, the former has the largest, while the latter has the smallest Kekulé count^33^ (Figure 4).

The stability of the unbranched catacondensed isomers of hexacene is biased by a steric hindrance, overcrowding, associated with a specific annelation leading to nonplanarity of the structure.^32^ Their symmetry and planarity are reflected in the PAH 3-digit code.^34^ The code used here assigns numbers 0, 1, and 2 to the nonterminal rings depending on annelation (number of H atoms in the edge, where the “clockwise” and “counterclockwise” angular annelations are differentiated by numbers 0 and 2, respectively). For instance, hexacene, benzo[c]picene, and hexahelicene are coded by 1111, 0202, and 0000, respectively (Figure 4). Notice that the original Balaban’s 3-digit code^34^ defines differently: 0 for linear annulations, and 1 or 2 for angular annulation. Still, the two 3-digit codes are equivalent.

**Figure 5:**
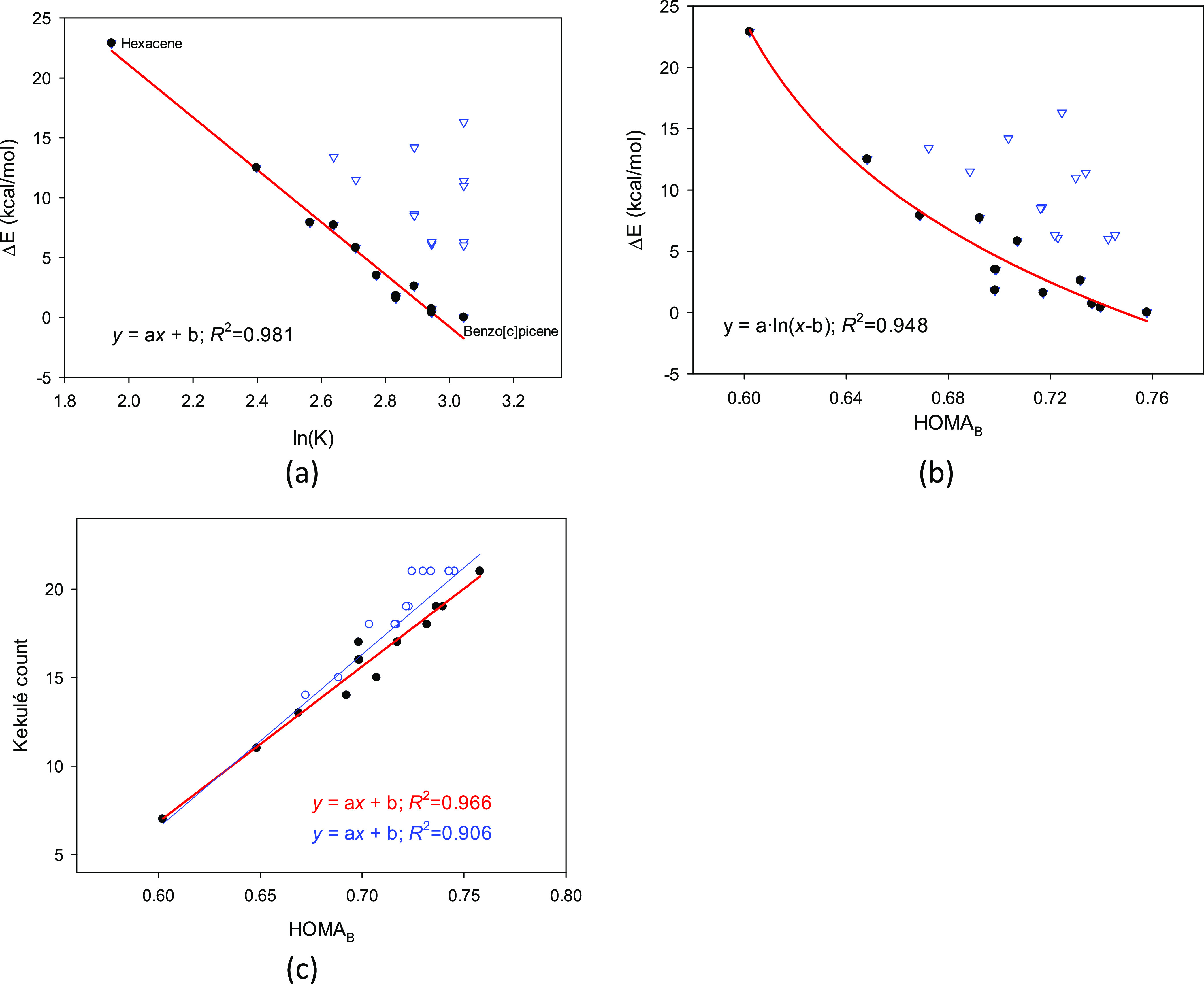
(a) Linear correlation between the total energy difference and the natural logarithm of the Kekulé count ln(K) for 25 unbranched catacondensed isomers of hexacene calculated at the B3LYP/D3/6-31G** level. (b) Logarithmic correlation between the total energy difference and the HOMA_B_ index calculated for entire molecules and (c) the linear correlations between the HOMA_B_ index calculated for entire molecules and the Kekulé count for all 25 structures (in blue) and only for the planar ones (in red). Blue empty cycles correspond to the nonplanar molecules (Figure 4).

A large number of nonplanar structures of the *C*_2_ or *C*_1_ point group symmetry (00 or 22 sequence present in the code) cause that correlation between the stabilization energy and molecular descriptors such as the Kekulé count occurs only after the addition of a term associated with the structure deviation from planarity.^32^ The Kekulé count *K* is the number of different perfect matchings of the structure with alternating single and double bonds (as in the Kekulé formula of benzene). Within the resonance theory stemming from the Hückel approximation of the molecular orbitals theory, the Kekulé count allows calculating the resonance energy (*RE*): *RE* = *A*·ln(*K*), where coefficient *A* = 0.1185 (eV).^33^ Still, the resonance theory of the conjugated polyhex hydrocarbons refers to planar structures.^35,36^ Consequently, the correlation of the total energy difference and the logarithm of the Kekulé count occurs only for planar hexacene isomers, while the nonplanar ones deviate substantially (Figure 5a).

However, the HOMA_B_ index calculated for the entire molecules of the unbranched catacondensed isomers of hexacene performs as well as the Kekulé count (Figure 5b). Indeed, there is a significant logarithmic correlation between HOMA_B_ and the total energy differences for planar structures (*R*^2^ = 0.948, Figure 5b). As for the Kekulé count, energies of the nonplanar ones deviate from it. Moreover, the linear correlation between the calculated HOMA_B_ index and the Kekulé count for all 25 structures is satisfactorily significant (*R*^2^ = 0.906, Figure 5c), and if only planar ones are considered, the correlation coefficient increases to *R*^2^ = 0.966 (Figure 5c). Finally, let us suggest that the HOMA_B_ index calculated for the entire molecules of the hexacene isomers can be understood as a similarity to a hypothetical isomer that would consist of six perfectly aromatic benzene rings. Let us also stress that, similarly to the classical HOMA index, the index can be applied to molecules composed of different numbers of rings and fused differently. So, it can be applied to how the topological indices are used in the Chemical Graph Theory. In fact, we have already demonstrated that the HOMA index is a topological index of the Structural Formula version of the Graph Theory.^37^

## 4 Final Remarks

### 4.1 HOMA Index for Molecules Containing Heteroatoms

A significant strength of the HOMA index is its ability to be generalized and parametrized for heteroatoms^3,7−11^ as follows
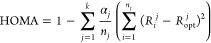
12where *j* = CC, CN or NC, CO or ON, ..., NN, etc., and thus *k* denotes the number of different (unordered) pairs of atoms in the cycle, α_j_ is a normalization factor for the *j*-th type of bonds, *n*_j_ is the number of the *j*-th kind of bonds, *R*_*i*_^*j*^ is the *i*-th bond of *j*-th type, and *R*_opt_^*j*^ is the optimal bond for the *j*-th type. The α_j_ and *R*_opt_^*j*^ parameters are found in the parametrization procedures referred to the experimental technique or computational level at which distances in a given ring are found or calculated.

Expression 12 can be rewritten to a more convenient form as follows
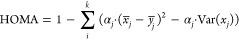
13where *j* indexes the kind of bond, *k* is the number of differentiated bonds, α_j_ is the *j*-th normalization factor, *x̅*_*j*_ and *y̅*_*j*_ are the means of all bonds of the *j*-th type and the appropriate *j*-th reference, respectively, and in the variance symbol, the *n*_j_ number of bonds of *j*-th-type is hidden.

The HOMA index defined in eqs 12 and 13 satisfies symmetricity and has a maximum axioms (8) and thus is a “chemical” similarity function as the primary HOMA index does.

### 4.2 Use of the HOMA Formula to Express Similarity Other Than Geometrical

The HOMA index was already studied as a function of the electron density properties in bond critical points (BCPs).^12,13^ In such a case, HOMA expressed similarity between electron density properties (electron density, potential, and kinetic forms of energy) measured in BCPs and was not a geometrical parameter anymore. However, the HOMA expression allows an even further deviation from the original intention. We can assume that *x* is an atom property such as partial charge, spin, or chemical shift. Then, *y* would be the appropriate property in the reference atom, and Var(x) would be the variance of this parameter in the examined moiety such as a ring. Since, for years, the ^13^C NMR spectra have been used for determining the aromaticity of the compounds, the comparison between the NICS class of indices and the HOMA-based NMR with the benzene C atom chemical shift as a reference would be especially intriguing. Such comparison is even more important because the chemical shift is observable. In contrast, most of the NICS indices are not, and only recently was it proven that the integral NICS index introduced by Stanger,^38^ INICS,^39,40^ was physically justified through its relation to the ring current via Ampère–Maxwell’s law as demonstrated by Berger et al.^41,42^

### 4.3 Use of the HOMA Formula to Study Acyclic Molecules

The HOMA index revealed both an increase in delocalization in polyene-like structures and an increase in insulation in unsaturated hydrocarbons,^12,13,16^ and a correlation with the boiling point in *n*-alkanes.^17^ However, juxtaposing the HOMA indices taken against ethane and ethene or acetylene references in analogy to that presented in Section 3.3.1 could uncover interesting new molecular features.

## 5 Conclusions

A close inspection of the definition of the HOMA aromaticity index revealed that it has the mathematical property of a similarity function. This property explains why the index, derived based on simple quantum chemical approximation, is such a good measure of geometrical aromaticity. It expresses a similarity to perfectly aromatic benzene.

An expression based on the transformation of the EN and GEO components of HOMA displays that to calculate the index, it is enough to know the arithmetical means of the CC bond lengths in the examined and reference rings and the variation of the CC bond lengths only in the examined moiety.

The similarity property of the HOMA function enables applying the index to evaluate similarity to other molecules, like cyclohexane. The HOMA-similarity to cyclohexane appeared to express a (relaxed)-saturicity not accompanied by strong structural strains or steric hindrances. A slight reformulation of the HOMA definition to allow the reference moiety to have some variation of the bond lengths, denoting a nonzero GEO factor, showed that similarity to the archetypical elusive cyclohexatriene ring can be better discriminated than using the classical HOMA index.

We demonstrated that HOMA can also be used to evaluate the properties of entire molecules, such as isomers of hexacene. The index calculated for all 25 unbranched catacondensed isomers of hexacene shows a significant quadratic correlation with the total energy differences of planar isomers from which the nonplanar ones deviate. Such an index could be interpreted as a similarity to a hypothetical isomer consisting of six perfectly aromatic benzene rings. Moreover, the HOMA index is significantly correlating with the Kekulé count (connected to the resonance energy within the frame of the Hückel approximation of the molecular orbitals theory) for all 25 isomers of hexacene (*R*^2^ > 0.9), but if only planar ones are considered, the correlation is much stronger (*R*^2^ > 0.96).

## Data Availability

All quantum chemical DFT calculations were done using commercially available Gaussian 09 software.^22^ The B3LYP functional,^18,19^ along with the D3 Grimme correction for dispersion forces,^20^ the 6-31G** Pople-type basis set,^21^ are accessible directly in the Gaussian program. Calculations of the HOMA indices and their components were done using commercial Microsoft Excel program, while correlations were performed using commercial SigmaPlot for Windows ver. 14.^43^

## Supporting Information Available

The XYZ coordinates of all molecules used, as well as six zipped files with original input and output Gaussian 09 files of all molecules calculated for Figures 1, 3, and 4. After decompression, the files can be automatically read by commercial Gaussian or GaussView software^44^ (PDF)
ad-Fig1-Gaussian16-input (ZIP)
ad-Fig1-Gaussian16-output (ZIP)
ad-Fig3-Central 6-membered-ring-Gaussian16-input (ZIP)
ad-Fig3-Central 6-membered-ring-Gaussian16-output (ZIP)
ad-Fig4-hexacene-Gaussian16-input (ZIP)
ad-Fig4-hexacene-Gaussian16-output (ZIP)
