# Supplementary material for: HOMA Index Establishes Similarity to a Reference Molecule
Source: J Chem Inf Model. 2023 Dec 6;63(24):7744–54. doi: 10.1021/acs.jcim.3c01551 (PMC10751799; doi:10.1021/acs.jcim.3c01551)
Supplement: Supplementary file 1 — ci3c01551_si_001.pdf [file ci3c01551_si_001.pdf]

Supporting Information to

The HOMA index establishes similarity to a reference molecule

Jan Cz. Dobrowolski* and Sławomir Ostrowski

Institute of Nuclear Chemistry and Technology, 16 Dorodna Street, 03-195 Warsaw, Poland

The article is dedicated to Professor Tadeusz Marek Krygowski, founder of the HOMA index and one of the most creative Polish organic physical chemists

Table of contents

#	Title	page
1	**Figure S1**. The relationships between indices calculated using benzene (B) and chair cyclohexane (C) reference molecules. (a) The HOMA indices truncated to the EN or GEO factors, 1-EN (black points) and 1-GEO (red points), rotated by 450 degrees. (b) The HOMA indices rotated by 450 degrees.	2
2	**Table S1.** The CC distances (Å) used to calculate HOMA indices amd their components. B and C stand for benzene and cyclohexane reference.	3
3	**Table S2.** The XYZ coordinates of the molecules presented in Fig. 1, Table 1, and Table S1.	5
4	**Table S3.** The XYZ coordinates of the molecules presented in Fig. 3 and Table 2.	15
5	**Table S4.** The XYZ coordinates of the hexacene molecules presented in Fig. 4.	19

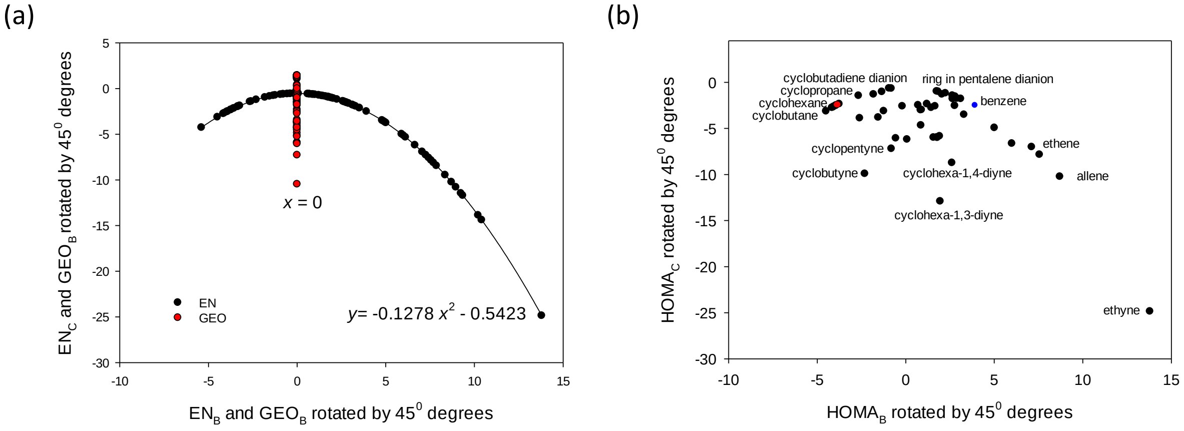

Figure S1. The relationships between indices calculated using benzene (B) and chair cyclohexane (C) reference molecules. The HOMA indices truncated to the EN or GEO factors, 1-EN (black points) and 1-GEO (red points), rotated by 45^0 degrees (a). The HOMA indices rotated by 45^0 degrees (b).

Seemingly, the curve in Fig. 2b is a deformed parabola, but the rotation of this curve by 45^0 degrees using pair of parametric equations shows that it is nothing but a parabola (Fig. S1). The half-straight line formed by 1-GEO (red points, Fig. 2b) overlaps with the OY axis in Fig. 2c. The parametric equations are the following: $X=t\cdot\cos(\theta)-f(t)\cdot\sin(\theta)$ and $Y=t\cdot\sin(\theta)+f(t)\cdot\cos(\theta)$, where $t=1-EN_B(A)$; $f(t)=1-EN_C(A)$; and $\sin(45^0)=\cos(45^0)=\sqrt{2}/2$, and thus reveal the quadratic dependence of Y on the independent variable X:

$$\begin{cases} X = \frac{\sqrt{2}}{2}\left(EN_C(A) - EN_B(A)\right) \\ Y = \frac{\sqrt{2}}{2}\left(2 - EN_C(A) - EN_B(A)\right) \end{cases} \Leftrightarrow \begin{cases} X = 2\sqrt{2}d(s-z) \\ Y = \sqrt{2}\left(1 - d^2 - \frac{X^2}{8d^2}\right) \end{cases}$$

where $$R_{opt,B} < R_{opt,C};\ s = \frac{R_{opt,B}+R_{opt,C}}{2}\ ;\ d = \frac{R_{opt,C}-R_{opt,B}}{2};\ z = R_{av,A}$$

$$R_{opt,B} = s - d;\ R_{opt,C} = s + d$$

Table S1. The CC distances (Å) used to calculate HOMA indices amd their components. B and C stand for benzene and cyclohexane reference. R_B =1.3962 Å and R_C=1.5428 Å. GEO(B)=GEO(C). The calculations were performed at the B3LYP/6-31G** level with the Grimmes D3 empirical dispersion correction.

Lp	molecule	R1	R2	R3	R4	R5	R6	R7	R8	R9	R10	R11	R12	average	variance	EN(B)	GEO(B)	HOMA(B)	EN(C)	HOMA(C)
1	benzene	1.3962	1.3962	1.3962	1.3962	1.3962	1.3962							1.3962	0.00000	0.00000	0.00000	1.00	5.53460	-4.53
2	cyclooctatetraene dication	1.4112	1.4112	1.4112	1.4112	1.4112	1.4112	1.4112	1.4112					1.4112	0.00000	0.05791	0.00000	0.94	4.45960	-3.46
3	side ring in triphenylene	1.4133	1.4212	1.4133	1.3828	1.4009	1.3828							1.4024	0.00023	0.00985	0.05861	0.93	5.07681	-4.14
4	cyclooctatetraene dianion	1.4170	1.4170	1.4170	1.4170	1.4170	1.4170							1.4170	0.00000	0.11138	0.00000	0.89	4.07503	-3.08
5	cyclopentadienyl anion	1.4200	1.4200	1.4200	1.4200	1.4200	1.4200							1.4200	0.00000	0.14585	0.00000	0.85	3.88291	-2.88
6	side ring in coronene	1.4275	1.4214	1.4214	1.4240	1.4240	1.3721							1.4151	0.00037	0.09163	0.09623	0.81	4.20129	-3.30
7	central ring in coronene	1.4275	1.4275	1.4274	1.4274	1.4275	1.4275							1.4275	0.00000	0.25177	0.00000	0.75	3.42490	-2.42
8	benzene anion radical	1.3978	1.4637	1.3978	1.4637	1.3978	1.3978							1.4198	0.00097	0.14291	0.24872	0.61	3.89815	-3.15
9	ring in pentalene dianion	1.4239	1.4375	1.4241	1.4374	1.4540								1.4354	0.00012	0.39539	0.03166	0.57	2.97085	-2.00
10	cycloheptatriene dianion	1.4100	1.4376	1.4620	1.3965	1.4414	1.4634	1.4072						1.4312	0.00062	0.31473	0.16051	0.52	3.20913	-2.37
11	central ring in triphenylene	1.4667	1.4212	1.4667	1.4212	1.4667	1.42122							1.4440	0.00052	0.58806	0.13349	0.28	2.51400	-1.65
12	cycloheptatriene anion	1.3987	1.3987	1.4590	1.3530	1.4589	1.5005	1.3532						1.4174	0.00276	0.11602	0.71150	0.17	4.04730	-3.76
13	ethene	1.3305												1.3305	0.00000	1.11202	0.00000	-0.11	11.60721	-10.61
14	benzyne	1.2511	1.3850	1.3847	1.4128	1.4072	1.4128							1.3756	0.00324	0.10946	0.83485	0.06	7.19991	-7.03
15	cyclooctatetraene	1.4735	1.3419	1.3419	1.4735	1.3419	1.4735	1.3419	1.4735					1.4077	0.00433	0.03402	1.11575	-0.15	4.70006	-4.82
16	C_6 cyclic molecule	1.3279	1.3279	1.3280	1.3280	1.3279	1.3280							1.3280	0.00000	1.20073	0.00000	-0.20	11.89003	-10.89
17	ring in pentalene	1.5035	1.3599	1.3621	1.4782	1.4694								1.4346	0.00374	0.38019	0.96357	-0.34	3.01305	-2.98
18	cyclohexa-1,3-diyn-5-ene	1.2808	1.3965	1.3346	1.2808	1.3964	1.4537							1.3571	0.00410	0.39350	1.05563	-0.45	8.87868	-8.93
19	ring in 1,4-dihydropentalene	1.5183	1.5008	1.3555	1.4559	1.3616								1.4384	0.00467	0.45914	1.20370	-0.66	2.80500	-3.01
20	cyclopentadiene	1.3548	1.5141	1.4748	1.3548	1.514								1.4425	0.00533	0.55219	1.37430	-0.93	2.58990	-2.96
21	cyclobutadiene dianion	1.5532	1.5532	1.5532	1.5532									1.5532	0.00000	6.35124	0.00000	-5.35	0.02814	0.97
22	allene	1.3064	1.3064											1.3064	0.00000	2.07857	0.00000	-1.08	14.39546	-13.40
23	central ring in 12*H*-coronene	1.4870	1.4870	1.4870	1.4870	1.4870	1.4870							1.4870	0.00000	2.12418	0.00000	-1.12	0.80095	0.20
24	cyclopentadienyl cation	1.57527	1.34943	1.34948	1.44986	1.44994								1.4348	0.00695	0.38365	1.79131	-1.17	3.00335	-3.79
25	off-center ring in 12*H*-coronene	1.3563	1.4870	1.5061	1.3563	1.5061	1.5262							1.4563	0.00513	0.93154	1.32237	-1.25	1.92446	-2.25
26	cyclohexa-1,3-diene	1.3432	1.5120	1.3432	1.5121	1.4674	1.5388							1.4528	0.00644	0.82478	1.66022	-1.48	2.08582	-2.75
27	cyclohexa-1,4-diene	1.3395	1.5107	1.3395	1.5107	1.5107	1.5107							1.4536	0.00651	0.84975	1.67845	-1.53	2.04660	-2.73

28	cyclobuta-1-yn-3-ene	1.4277	1.6027	1.4339	1.4277									1.4730	0.00561	1.51958	1.44667	-1.97	1.25373	-1.70
29	ring in bicyclo[1.1.0]butane	1.5149	1.5149	1.5094										1.5130	0.00001	3.51741	0.00175	-2.52	0.22747	0.77
30	cyclopropene	1.5278	1.3046	1.5279										1.4534	0.01108	0.84404	2.85446	-2.70	2.05549	-3.91
31	cyclopenta-1-yn-3-ene	1.2423	1.5299	1.4732	1.3678	1.5544								1.4335	0.01327	0.35873	3.42018	-2.78	3.07467	-5.49
32	cyclopropane	1.5194	1.5193	1.5193										1.5193	0.00000	3.90657	0.00000	-2.91	0.14131	0.86
33	cyclopentadiyne	1.2784	1.5744	1.4730	1.3181	1.5362								1.4360	0.01386	0.40841	3.57181	-2.98	2.93554	-5.51
34	cyclohexa-1-yn-3-ene	1.2263	1.4802	1.4487	1.3549	1.5224	1.5871							1.4366	0.01383	0.42040	3.56314	-2.98	2.90372	-5.47
35	cyclopentene	1.5517	1.5519	1.5124	1.5124	1.3350								1.4927	0.00653	2.39827	1.68181	-3.08	0.64606	-1.33
36	cyclohexa-1-yn-4-ene	1.2260	1.4814	1.4815	1.3441	1.5537	1.5538							1.4401	0.01405	0.49604	3.62099	-3.12	2.71627	-5.34
37	cyclobutene	1.5301	1.3478	1.5865	1.5300									1.4986	0.00811	2.70165	2.09029	-3.79	0.50231	-1.59
38	cycloheptane	1.5391	1.5406	1.5354	1.5391	1.5419	1.5407	1.5419						1.5398	0.00000	5.31434	0.00113	-4.32	0.00222	1.00
39	cyclohexa-1,4-diyne	1.2202	1.5206	1.5206	1.2202	1.5205	1.5205							1.4204	0.02005	0.15121	5.16603	-4.32	3.85554	-8.02
40	cyclobutadiene	1.5926	1.3435	1.3435	1.5926									1.4681	0.01551	1.32999	3.99762	-4.33	1.43799	-4.44
41	chair cyclohexane	1.5427	1.5427	1.5427	1.5429	1.5427	1.5429							1.5428	0.00000	5.53510	0.00000	-4.54	0.00000	1.00
42	adamantane	1.5433	1.5433	1.5433	1.5433	1.5433	1.5433	1.5433	1.5433	1.5433	1.5433	1.5433	1.5433	1.5433	0.00000	5.57546	0.00000	-4.58	0.00008	1.00
43	cyclohexene	1.5577	1.5966	1.3554	1.5011	1.5011	1.5966							1.5181	0.00682	3.82765	1.75846	-4.59	0.15680	-0.92
44	boat cyclohexane	1.5482	1.5482	1.5481	1.5345	1.5481	1.5345							1.5436	0.00004	5.59823	0.01067	-4.61	0.00019	0.99
45	cyclohexyne	1.2207	1.4795	1.4795	1.5765	1.5766	1.5467							1.4799	0.01505	1.80565	3.87966	-4.69	1.01741	-3.90
46	neopentane	1.5395	1.5395	1.5395	1.5395									1.5395	0.00000	5.29187	0.00000	-4.29	0.00270	1.00
47	cyclopentane	1.5420	1.5576	1.5360	1.5400	1.5551								1.5461	0.00007	5.79284	0.01903	-4.81	0.00296	0.98
48	cyclooctane	1.5427	1.5427	1.5520	1.5427	1.5520	1.5520	1.5427	1.5520					1.5474	0.00002	5.88672	0.00557	-4.89	0.00545	0.99
49	cyclobutane	1.5537	1.5537	1.5537	1.5537									1.5537	0.00000	6.39176	0.00000	-5.39	0.03090	0.97
50	cyclopentyne	1.5896	1.5895	1.5090	1.5090	1.2259								1.4846	0.01803	2.01336	4.64609	-5.66	0.87139	-4.52
51	cyclohexa-1,3-diyne	1.2470	1.4713	1.2470	1.4714	1.3804	1.7803							1.4329	0.03259	0.34690	8.39747	-7.74	3.10967	-10.51
52	ethyne	1.2112												1.2112	0.00000	8.82074	0.00000	-7.82	28.32778	-27.33

Table S2. The XYZ coordinates of the molecules presented in Fig. 1, Table 1, and Table S1. The calculations were performed at the B3LYP/6-31G** level with the Grimmes D3 empirical dispersion correction.

	1				2				3		
	x	y	z		x	y	z		x	y	z
C	0.00000	1.39620	0.00000	C	0.00000	1.85140	0.00000	C	0.00000	1.38390	2.49970
C	-1.20910	0.69810	0.00000	H	0.00000	2.95430	0.00000	C	0.00000	0.71050	1.25710
C	-1.20910	-0.69810	0.00000	C	-1.30920	1.30920	0.00000	C	0.00000	1.44390	-0.01320
C	0.00000	-1.39620	0.00000	H	-2.08900	2.08900	0.00000	C	0.00000	2.85670	-0.05130
C	1.20910	-0.69810	0.00000	C	1.30920	1.30920	0.00000	C	0.00000	-0.71050	1.25710
C	1.20910	0.69810	0.00000	H	2.08900	2.08900	0.00000	C	0.00000	0.73340	-1.24390
H	0.00000	2.48230	0.00000	C	1.85140	0.00000	0.00000	C	0.00000	-0.73340	-1.24390
H	-2.14970	1.24110	0.00000	H	2.95430	0.00000	0.00000	C	0.00000	-1.44390	-0.01320
H	-2.14970	-1.24110	0.00000	C	-1.85140	0.00000	0.00000	C	0.00000	-1.47280	-2.44840
H	0.00000	-2.48230	0.00000	H	-2.95430	0.00000	0.00000	C	0.00000	1.47280	-2.44840
H	2.14970	-1.24110	0.00000	C	-1.30920	-1.30920	0.00000	C	0.00000	2.85560	-2.45750
H	2.14970	1.24110	0.00000	H	-2.08900	-2.08900	0.00000	C	0.00000	3.55610	-1.24420
				C	0.00000	-1.85140	0.00000	H	0.00000	4.64190	-1.23630
				H	0.00000	-2.95430	0.00000	H	0.00000	3.39170	-3.40180
				C	1.30920	-1.30920	0.00000	C	0.00000	0.70050	3.70170
				H	2.08900	-2.08900	0.00000	C	0.00000	-0.70050	3.70170
								C	0.00000	-1.38390	2.49970
								H	0.00000	1.25020	4.63820
								H	0.00000	-1.25020	4.63820
								C	0.00000	-2.85670	-0.05130
								C	0.00000	-2.85560	-2.45750
								H	0.00000	-3.39170	-3.40180
								C	0.00000	-3.55610	-1.24420
								H	0.00000	-4.64190	-1.23630
								H	0.00000	0.95530	-3.39960
								H	0.00000	-0.95530	-3.39960
								H	0.00000	-3.42170	0.87250
								H	0.00000	-2.46650	2.52710
								H	0.00000	2.46650	2.52710
								H	0.00000	3.42170	0.87250

	4				5				6		
	x	y	z		x	y	z		x	y	z
C	0.00000	1.84380	0.00000	C	0.00000	1.20790	0.00000	C	1.24830	3.53420	0.00000
H	0.00000	2.93370	0.00000	H	0.00000	2.29690	0.00000	C	0.00000	2.84890	0.00000
C	1.30380	1.30380	0.00000	C	1.14880	0.37330	0.00000	C	0.00000	1.42750	0.00000
H	2.07440	2.07440	0.00000	H	2.18450	0.70980	0.00000	C	1.23620	0.71370	0.00000
C	-1.30380	1.30380	0.00000	C	0.71000	-0.97720	0.00000	C	2.46720	1.42440	0.00000
H	-2.07440	2.07440	0.00000	H	1.35010	-1.85820	0.00000	C	2.43660	2.84810	0.00000
C	-1.84380	0.00000	0.00000	C	-1.14880	0.37330	0.00000	C	-1.23620	0.71370	0.00000
H	-2.93370	0.00000	0.00000	H	-2.18450	0.70980	0.00000	C	1.23620	-0.71370	0.00000
C	1.84380	0.00000	0.00000	C	-0.71000	-0.97720	0.00000	C	0.00000	-1.42750	0.00000

H	2.93370	0.00000	0.00000	H	-1.35010	-1.85820	0.00000	C	-1.23620	-0.71370	0.00000
C	1.30380	-1.30380	0.00000					C	0.00000	-2.84890	0.00000
H	2.07440	-2.07440	0.00000					C	1.24830	-3.53420	0.00000
C	0.00000	-1.84380	0.00000					C	2.43660	-2.84810	0.00000
H	0.00000	-2.93370	0.00000					C	2.46720	-1.42440	0.00000
C	-1.30380	-1.30380	0.00000					C	3.68480	-0.68610	0.00000
H	-2.07440	-2.07440	0.00000					C	3.68480	0.68610	0.00000
								H	4.62460	1.23190	0.00000
								H	4.62460	-1.23190	0.00000
								H	1.24550	4.62100	0.00000
								H	3.37910	3.38910	0.00000
								H	1.24550	-4.62100	0.00000
								H	3.37910	-3.38910	0.00000
								C	-1.24830	3.53420	0.00000
								C	-2.43660	2.84810	0.00000
								C	-2.46720	1.42440	0.00000
								H	-1.24550	4.62100	0.00000
								H	-3.37910	3.38910	0.00000
								C	-3.68480	0.68610	0.00000
								C	-3.68480	-0.68610	0.00000
								C	-2.46720	-1.42440	0.00000
								H	-4.62460	1.23190	0.00000
								H	-4.62460	-1.23190	0.00000
								C	-1.24830	-3.53420	0.00000
								H	-1.24550	-4.62100	0.00000
								C	-2.43660	-2.84810	0.00000
								H	-3.37910	-3.38910	0.00000
7				8				9			
	x	y	z		x	y	z		x	y	z
C	1.24830	3.53420	0.00000	C	0.73170	1.22890	0.00000	C	-1.36870	-1.16630	0.00000
C	0.00000	2.84890	0.00000	C	1.39800	0.00020	0.00000	C	-2.18560	0.00000	0.00040
C	0.00000	1.42750	0.00000	C	0.73200	-1.22880	0.00000	C	-1.36860	1.16640	0.00010
C	1.23620	0.71370	0.00000	C	-0.73170	-1.22890	0.00000	C	0.00000	0.72700	-0.00010
C	2.46720	1.42440	0.00000	C	-1.39800	-0.00020	0.00000	C	0.00000	-0.72700	0.00000
C	2.43660	2.84810	0.00000	C	-0.73200	1.22880	0.00000	C	1.36870	1.16630	-0.00040
C	-1.23620	0.71370	0.00000	H	1.28990	2.16430	0.00000	C	2.18560	0.00000	0.00030
C	1.23620	-0.71370	0.00000	H	2.49370	0.00030	0.00000	C	1.36860	-1.16640	-0.00020
C	0.00000	-1.42750	0.00000	H	1.29050	-2.16400	0.00000	H	-1.73400	-2.19900	-0.00020
C	-1.23620	-0.71370	0.00000	H	-1.28990	-2.16430	0.00000	H	-3.28430	0.00000	0.00050
C	0.00000	-2.84890	0.00000	H	-1.29050	2.16400	0.00000	H	-1.73400	2.19900	0.00010
C	1.24830	-3.53420	0.00000	H	-2.49370	-0.00030	0.00000	H	1.73410	2.19900	-0.00080
C	2.43660	-2.84810	0.00000					H	3.28430	0.00000	0.00070
C	2.46720	-1.42440	0.00000					H	1.73400	-2.19910	-0.00030
C	3.68480	-0.68610	0.00000								
C	3.68480	0.68610	0.00000								
H	4.62460	1.23190	0.00000								

H	4.62460	-1.23190	0.00000								
H	1.24550	4.62100	0.00000								
H	3.37910	3.38910	0.00000								
H	1.24550	-4.62100	0.00000								
H	3.37910	-3.38910	0.00000								
C	-1.24830	3.53420	0.00000								
C	-2.43660	2.84810	0.00000								
C	-2.46720	1.42440	0.00000								
H	-1.24550	4.62100	0.00000								
H	-3.37910	3.38910	0.00000								
C	-3.68480	0.68610	0.00000								
C	-3.68480	-0.68610	0.00000								
C	-2.46720	-1.42440	0.00000								
H	-4.62460	1.23190	0.00000								
H	-4.62460	-1.23190	0.00000								
C	-1.24830	-3.53420	0.00000								
H	-1.24550	-4.62100	0.00000								
C	-2.43660	-2.84810	0.00000								
H	-3.37910	-3.38910	0.00000								
	10				11				12		
	x	y	z		x	y	z		x	y	z
C	-0.89340	1.36870	0.00190	C	0.00000	1.38390	2.49970	C	-0.00140	1.59540	-0.00010
C	0.50050	1.58010	0.02490	C	0.00000	0.71050	1.25710	C	1.25680	1.02160	-0.20970
C	1.54740	0.56000	-0.00620	C	0.00000	1.44390	-0.01320	C	1.56900	-0.38020	0.04730
C	-1.65820	0.15220	-0.04130	C	0.00000	2.85670	-0.05130	C	-1.25860	1.01940	0.20990
C	1.42190	-0.83080	-0.00730	C	0.00000	-0.71050	1.25710	C	0.73880	-1.44480	0.13750
C	-1.12580	-1.18660	0.00260	C	0.00000	0.73340	-1.24390	C	-1.56830	-0.38290	-0.04700
C	0.20510	-1.64330	0.02180	C	0.00000	-0.73340	-1.24390	C	-0.73620	-1.44610	-0.13790
H	-1.50170	2.29250	-0.01030	C	0.00000	-1.44390	-0.01320	H	-0.00240	2.69490	-0.00040
H	0.84220	2.61840	-0.11730	C	0.00000	-1.47280	-2.44840	H	2.10500	1.69470	-0.32930
H	2.57910	0.94970	0.04250	C	0.00000	1.47280	-2.44840	H	2.62730	-0.58610	0.26160
H	-2.73410	0.25040	0.17900	C	0.00000	2.85560	-2.45750	H	-2.10810	1.69110	0.32890
H	2.36540	-1.40070	0.04740	C	0.00000	3.55610	-1.24420	H	1.16490	-2.39830	0.46240
H	-1.89090	-1.98500	-0.01270	H	0.00000	4.64190	-1.23630	H	-2.62640	-0.59070	-0.26050
H	0.35420	-2.72780	-0.10670	H	0.00000	3.39170	-3.40180	H	-1.16080	-2.40030	-0.46240
				C	0.00000	0.70050	3.70170				
				C	0.00000	-0.70050	3.70170				
				C	0.00000	-1.38390	2.49970				
				H	0.00000	1.25020	4.63820				
				H	0.00000	-1.25020	4.63820				
				C	0.00000	-2.85670	-0.05130				
				C	0.00000	-2.85560	-2.45750				
				H	0.00000	-3.39170	-3.40180				
				C	0.00000	-3.55610	-1.24420				
				H	0.00000	-4.64190	-1.23630				
				H	0.00000	0.95530	-3.39960				

	x	y	z
H	0.00000	-0.95530	-3.39960
H	0.00000	-3.42170	0.87250
H	0.00000	-2.46650	2.52710
H	0.00000	2.46650	2.52710
H	0.00000	3.42170	0.87250

13

	x	y	z
C	0.00000	0.66530	0.00000
H	0.92350	1.23830	0.00000
H	-0.92350	1.23830	0.00000
C	0.00000	-0.66530	0.00000
H	-0.92350	-1.23830	0.00000
H	0.92350	-1.23830	0.00000

14

	x	y	z
C	0.62660	-1.23750	0.00030
C	-0.62450	-1.23740	-0.00020
C	-1.46190	-0.13460	0.00010
C	-0.70440	1.05800	0.00000
H	-2.54650	-0.13590	0.00010
H	-1.23040	2.00950	-0.00010
C	1.46180	-0.13270	-0.00010
H	2.54650	-0.13230	0.00000
C	0.70280	1.05890	0.00000
H	1.22760	2.01110	0.00010

15

	x	y	z
C	-1.56410	-0.67890	0.37540
C	-0.66300	-1.57100	-0.37520
C	0.66300	1.57100	-0.37520
C	0.67890	-1.56410	-0.37530
C	1.56410	0.67890	0.37530
C	1.57100	-0.66300	0.37520
H	-2.35340	-1.18640	0.93180
H	-1.16240	-2.36540	-0.93150
H	1.16230	2.36550	-0.93150
H	1.18630	-2.35350	-0.93170
H	2.35350	1.18640	0.93160
H	2.36550	-1.16240	0.93140
C	-1.57100	0.66300	0.37520
H	-2.36540	1.16240	0.93160
C	-0.67890	1.56410	-0.37530
H	-1.18640	2.35340	-0.93170

16

	x	y	z
C	0.36330	1.43240	0.00020
C	-0.78320	0.76250	-0.00010
C	-1.42230	-0.40160	0.00010
C	-0.26870	-1.05940	-0.00010
C	1.05890	-1.03090	0.00020
C	1.05200	0.29710	-0.00030

17

	x	y	z
C	-1.30500	1.11360	-0.00010
C	-2.13190	-0.14210	-0.00010
C	-1.30840	-1.22710	0.00000
C	0.07920	-0.71750	0.00000
C	0.00540	0.75000	0.00000
C	1.38970	-1.08110	0.00000
C	2.21660	0.17440	0.00010
C	1.39310	1.25950	0.00000
H	-1.72250	2.11300	-0.00020
H	-3.21360	-0.15130	-0.00010
H	-1.60710	-2.26620	0.00000
H	1.80700	-2.08060	0.00020
H	3.29820	0.18380	0.00020
H	1.69190	2.29860	0.00000

18

	x	y	z
C	-0.40510	-0.09630	-0.00610
C	0.87460	-0.04360	-0.00590
C	1.54190	1.11220	-0.00650
C	0.94770	2.24680	-0.00750
C	-0.44630	2.32890	-0.00780
C	-1.17320	1.07000	-0.00700
H	-1.02140	3.24670	-0.00850
H	-2.25550	1.10910	-0.00730

19

	x	y	z
C	-1.43340	1.15930	-0.00010
C	-2.20730	-0.14700	-0.00010
C	-1.35020	-1.19710	-0.00010
C	0.01100	-0.68070	0.00000
C	-0.01100	0.68070	0.00000
C	1.43340	-1.15930	0.00010

20

	x	y	z
C	-0.34610	-0.50620	-1.18580
H	-0.15380	-0.23700	-2.21630
C	-1.08940	-1.54630	-0.73740
H	-1.61000	-2.27450	-1.34730
C	-0.34620	-0.50610	1.18580
H	-0.15390	-0.23690	2.21630

21

	x	y	z
C	1.09820	-0.00060	0.00300
H	1.65390	0.00060	1.02710
H	1.65970	-0.00230	-1.01750
C	0.00050	1.09820	-0.00190
H	0.00300	1.65480	-1.02560
H	-0.00140	1.65880	1.01910

C	2.20730	0.14700	0.00010	C	-1.08940	-1.54630	0.73740	C	-1.09830	0.00050	-0.00230
C	1.35020	1.19710	0.00010	H	-1.61000	-2.27440	1.34740	H	-1.65850	0.00220	1.01920
H	-1.67610	1.77330	-0.87890	C	0.20170	0.25940	0.00000	H	-1.65500	-0.00060	-1.02550
H	-1.67610	1.77330	0.87860	H	1.30260	0.28610	0.00000	C	-0.00060	-1.09830	0.00130
H	-3.28940	-0.19890	-0.00010	H	-0.12880	1.30990	0.00000	H	0.00160	-1.65760	-1.02080
H	-1.63030	-2.24480	0.00000					H	-0.00330	-1.65590	1.02400
H	1.67600	-1.77330	0.87890								
H	1.67610	-1.77340	-0.87850								
H	3.28940	0.19890	0.00010								
H	1.63030	2.24480	0.00010								
		22				23				24	
	x	y	z		x	y	z		x	y	z
C	-1.30640	0.00000	0.00000	C	0.00000	2.84330	0.00000	C	-0.10310	-0.03730	1.16030
C	0.00000	0.00000	0.00000	C	0.00000	1.48700	0.00000	H	-0.15510	0.03140	2.24120
H	-1.87330	0.65640	-0.65590	C	-1.28780	0.74350	0.00000	C	0.23880	1.18040	0.22120
H	-1.87320	-0.65630	0.65610	C	-2.46240	1.42170	-0.00010	H	0.44840	2.18050	0.58420
C	1.30640	0.00000	0.00000	C	1.28780	0.74350	0.00000	C	0.21620	0.73960	-1.05410
H	1.87330	-0.65600	-0.65640	C	-1.28780	-0.74350	0.00000	H	0.40270	1.29590	-1.96190
H	1.87310	0.65620	0.65630	C	0.00000	-1.48700	0.00000	C	-0.30530	-1.11900	0.37930
				C	1.28780	-0.74350	0.00000	H	-0.55810	-2.12670	0.67760
				C	0.00000	-2.84330	0.00000	C	-0.11550	-0.67010	-0.98630
				C	-2.46240	-1.42170	0.00010	H	-0.21120	-1.31590	-1.85860
				C	2.46240	1.42170	0.00010				
				C	2.46240	-1.42170	-0.00010				
				C	-3.78760	-0.73550	0.20340				
				H	-4.58130	-1.27600	-0.32790				
				H	-4.03760	-0.83070	1.27350				
				C	-3.78760	0.73550	-0.20340				
				H	-4.58130	1.27600	0.32790				
				H	-4.03760	0.83070	-1.27350				
				C	-2.53080	-2.91240	-0.20310				
				H	-3.39560	-3.32950	0.32840				
				H	-2.73860	-3.08120	-1.27320				
				C	-1.25680	-3.64790	0.20320				
				H	-1.18560	-4.60540	-0.32830				
				H	-1.29920	-3.91230	1.27330				
				C	1.25680	-3.64790	-0.20320				
				H	1.18560	-4.60540	0.32830				
				H	1.29920	-3.91230	-1.27330				
				C	2.53080	-2.91240	0.20310				
				H	3.39560	-3.32950	-0.32840				
				H	2.73860	-3.08120	1.27320				
				C	3.78760	-0.73550	-0.20340				
				H	4.58130	-1.27600	0.32790				
				H	4.03760	-0.83070	-1.27350				
				C	3.78760	0.73550	0.20340				

				H	4.58130	1.27600	-0.32790				
				H	4.03760	0.83070	1.27350				
				C	2.53080	2.91240	-0.20310				
				H	3.39560	3.32950	0.32840				
				H	2.73860	3.08120	-1.27320				
				C	1.25680	3.64790	0.20320				
				H	1.18560	4.60540	-0.32830				
				H	1.29920	3.91230	1.27330				
				C	-1.25680	3.64790	-0.20320				
				H	-1.18560	4.60540	0.32830				
				H	-1.29920	3.91230	-1.27330				
				C	-2.53080	2.91240	0.20310				
				H	-3.39560	3.32950	-0.32840				
				H	-2.73860	3.08120	1.27320				
25				26				27			
	x	y	z		x	y	z		x	y	z
C	0.00000	2.84330	0.00000	C	0.11160	-1.42500	-0.06560	C	-0.83550	-0.14770	0.02220
C	0.00000	1.48700	0.00000	C	0.11260	1.42490	0.06560	C	1.34540	1.11130	0.02210
C	-1.28780	0.74350	0.00000	C	1.25850	0.72540	0.10690	C	0.67570	2.27140	0.02130
C	-2.46240	1.42170	-0.00010	C	1.25800	-0.72630	-0.10690	C	-1.50520	1.01240	0.02140
C	1.28780	0.74350	0.00000	H	0.11210	-2.50510	-0.18880	H	-1.38060	-1.08970	0.02280
C	-1.28780	-0.74350	0.00000	H	0.11390	2.50500	0.18880	H	2.43370	1.11230	0.02260
C	0.00000	-1.48700	0.00000	H	2.20820	1.22510	0.28010	H	1.22080	3.21340	0.02110
C	1.28780	-0.74350	0.00000	H	2.20730	-1.22670	-0.28020	H	-2.59360	1.01140	0.02130
C	0.00000	-2.84330	0.00000	C	-1.19310	0.72920	-0.24660	C	0.67230	-0.24120	0.02240
C	-2.46240	-1.42170	0.00010	H	-2.04020	1.27260	0.18560	H	1.00980	-0.82570	0.89560
C	2.46240	1.42170	0.00010	H	-1.34210	0.74780	-1.33900	H	1.01000	-0.82610	-0.85050
C	2.46240	-1.42170	-0.00010	C	-1.19360	-0.72840	0.24660	C	-0.83210	2.36490	0.02060
C	-3.78760	-0.73550	0.20340	H	-2.04110	-1.27120	-0.18570	H	-1.16940	2.94890	-0.85310
H	-4.58130	-1.27600	-0.32790	H	-1.34270	-0.74680	1.33900	H	-1.17010	2.95040	0.89300
H	-4.03760	-0.83070	1.27350								
C	-3.78760	0.73550	-0.20340								
H	-4.58130	1.27600	0.32790								
H	-4.03760	0.83070	-1.27350								
C	-2.53080	-2.91240	-0.20310								
H	-3.39560	-3.32950	0.32840								
H	-2.73860	-3.08120	-1.27320								
C	-1.25680	-3.64790	0.20320								
H	-1.18560	-4.60540	-0.32830								
H	-1.29920	-3.91230	1.27330								
C	1.25680	-3.64790	-0.20320								
H	1.18560	-4.60540	0.32830								
H	1.29920	-3.91230	-1.27330								
C	2.53080	-2.91240	0.20310								
H	3.39560	-3.32950	-0.32840								
H	2.73860	-3.08120	1.27320								

C	3.78760	-0.73550	-0.20340								
H	4.58130	-1.27600	0.32790								
H	4.03760	-0.83070	-1.27350								
C	3.78760	0.73550	0.20340								
H	4.58130	1.27600	-0.32790								
H	4.03760	0.83070	1.27350								
C	2.53080	2.91240	-0.20310								
H	3.39560	3.32950	0.32840								
H	2.73860	3.08120	-1.27320								
C	1.25680	3.64790	0.20320								
H	1.18560	4.60540	-0.32830								
H	1.29920	3.91230	1.27330								
C	-1.25680	3.64790	-0.20320								
H	-1.18560	4.60540	0.32830								
H	-1.29920	3.91230	-1.27330								
C	-2.53080	2.91240	0.20310								
H	-3.39560	3.32950	-0.32840								
H	-2.73860	3.08120	1.27320								
	28				29				30		
	x	y	z		x	y	z		x	y	z
C	0.02310	0.28930	0.49700	C	1.35970	-0.09790	0.25890	C	0.48850	0.01370	0.00000
C	1.36220	0.20100	0.00970	H	1.11830	-0.52330	1.23490	C	2.01000	-0.12440	0.00000
C	1.36220	1.63480	-0.00990	H	2.20060	-0.58110	-0.23390	C	1.14970	1.13830	0.00000
C	0.02310	1.54640	-0.49710	C	-0.29790	1.48650	0.29470	H	-0.45060	-0.51060	0.00000
H	1.98740	-0.57190	-0.41140	H	-0.82940	2.31500	-0.16840	H	2.52670	-0.42900	-0.91190
H	1.98720	2.40760	0.41160	H	-0.69000	1.20500	1.27390	H	2.52670	-0.42900	0.91190
				C	0.24460	0.41520	-0.62880	H	1.15350	2.21380	0.00000
				H	0.03400	0.21840	-1.66770				
				C	1.19040	1.38990	0.02960				
				H	1.87250	2.11300	-0.38780				
	31				32				33		
	x	y	z		x	y	z		x	y	z
C	-0.37490	-0.54410	-1.16860	C	0.35650	-0.08510	0.00000	C	-0.35580	-0.51750	-1.23350
C	-1.04950	-1.48830	-0.72500	C	1.87590	-0.08500	0.00000	C	-0.97480	-1.38420	-0.52640
C	-0.35120	-0.51540	1.19090	C	1.11610	1.23070	0.00000	C	-0.43140	-0.62750	0.92090
H	-0.16170	-0.25230	2.22700	H	-0.15800	-0.38220	-0.90850	C	-1.19820	-1.69930	0.89510
C	-1.10350	-1.56720	0.74510	H	-0.15800	-0.38220	0.90850	C	0.25580	0.33490	-0.05960
H	-1.62560	-2.29850	1.34510	H	2.39040	-0.38210	-0.90850	H	1.34460	0.30680	-0.01840
C	0.21470	0.27740	-0.02050	H	2.39040	-0.38210	0.90850	H	-0.12220	1.35630	-0.01630
H	1.31040	0.28880	-0.03100	H	1.11610	1.82480	0.90850				
H	-0.12800	1.31820	-0.02900	H	1.11610	1.82480	-0.90850				
	34				35				36		
	x	y	z		x	y	z		x	y	z
C	0.57240	-1.27300	-0.13190	C	0.00000	-1.22540	-0.14270	C	1.78340	0.39240	-0.00010
C	-0.65270	-1.29660	-0.08220	C	1.23400	-0.31870	0.10830	C	2.39640	1.45410	0.00010
C	-1.54200	-0.16070	0.05020	C	-1.23410	-0.31860	0.10830	C	0.36540	2.69490	-0.00130

C	-0.85910	1.00920	0.07530	C	0.66750	1.07470	-0.04910	C	-0.30660	1.53090	-0.00120
H	-2.61600	-0.22130	0.19070	C	-0.66750	1.07480	-0.04910	H	-0.19140	3.63050	-0.00180
H	-1.39910	1.94360	0.21630	H	0.00000	-1.55000	-1.18870	H	-1.39530	1.54530	-0.00170
C	1.50460	-0.16930	0.19030	H	0.00000	-2.12600	0.47780	C	0.32860	0.11290	-0.00040
H	1.76090	-0.19400	1.25810	H	2.05780	-0.52950	-0.58410	H	-0.00900	-0.45240	-0.88010
H	2.44360	-0.14550	-0.37050	H	1.64080	-0.45360	1.12170	H	-0.00940	-0.45150	0.87970
C	0.64010	1.11240	-0.16860	H	-1.64080	-0.45360	1.12170	C	1.91100	2.85380	-0.00080
H	0.77600	1.30150	-1.24450	H	-2.05780	-0.52950	-0.58410	H	2.23100	3.42920	0.87890
H	1.05470	1.98420	0.35090	H	1.29290	1.96060	-0.10930	H	2.23160	3.42820	-0.88090
				H	-1.29280	1.96070	-0.10930				
		37				38				39	
	x	y	z		x	y	z		x	y	z
C	-0.00660	0.48330	0.00000	C	0.31060	-1.52060	-0.40540	C	-0.61040	-1.14250	-0.00010
C	1.51880	0.36400	0.00000	C	1.54480	-0.75960	0.11080	C	0.60980	-1.14250	0.00010
C	1.51870	1.95050	-0.00010	C	1.54480	0.75970	-0.11080	C	0.61040	1.14250	0.00000
C	-0.00660	1.83110	-0.00020	C	-0.96730	-1.24530	0.40990	C	-0.60980	1.14240	0.00000
H	-0.79020	-0.26550	-0.00020	C	0.31060	1.52060	0.40550	C	-1.61350	0.00030	0.00000
H	1.96440	-0.09430	-0.89040	C	-1.77890	0.00000	0.00000	H	-2.26380	0.00050	-0.88100
H	1.96440	-0.09420	0.89050	C	-0.96730	1.24530	-0.40990	H	-2.26360	0.00050	0.88120
H	1.96440	2.40870	-0.89050	H	0.14230	-1.29330	-1.46740	C	1.61350	-0.00020	0.00000
H	1.96430	2.40880	0.89030	H	1.64430	-0.96400	1.18670	H	2.26380	-0.00030	0.88100
H	-0.79030	2.57990	-0.00020	H	1.64430	0.96400	-1.18670	H	2.26360	-0.00040	-0.88120
				H	0.14230	1.29330	1.46740				
				H	-0.68150	-1.16220	1.46700				
				H	-2.44160	0.26080	0.83530				
				H	0.53280	-2.59390	-0.35780				
				H	2.44580	-1.17440	-0.35970				
				H	2.44570	1.17450	0.35970				
				H	-1.63370	-2.11480	0.35420				
				H	0.53270	2.59390	0.35780				
				H	-2.44150	-0.26090	-0.83540				
				H	-1.63370	2.11470	-0.35410				
				H	-0.68160	1.16220	-1.46700				
		40				41				42	
	x	y	z		x	y	z		x	y	z
C	-0.04460	0.48550	0.00000	H	-2.49030	-0.26290	-0.13660	C	0.00000	0.00000	1.78050
C	1.54800	0.48550	0.00000	C	-1.46230	-0.15440	0.23350	H	0.62410	0.62410	2.43370
C	1.54790	1.82900	0.00000	C	0.86490	-1.18910	0.23350	H	-0.62410	-0.62410	2.43370
C	-0.04470	1.82900	-0.00030	C	0.59750	1.34350	0.23350	C	0.89250	-0.89250	0.89250
H	-0.80330	-0.28500	-0.00030	C	1.46230	0.15440	-0.23350	H	1.52610	-1.52610	1.52610
H	2.30660	-0.28500	0.00040	C	-0.86490	1.18910	-0.23350	C	-0.89250	0.89250	0.89250
H	2.30660	2.59950	-0.00030	C	-0.59750	-1.34350	-0.23350	H	-1.52610	1.52610	1.52610
H	-0.80330	2.59940	-0.00010	H	0.89780	-1.23450	1.33300	C	0.00000	1.78050	0.00000
				H	0.62020	1.39470	1.33300	H	0.62410	2.43370	0.62410
				H	1.51810	0.16020	-1.33300	H	-0.62410	2.43370	-0.62410
				H	-0.89780	1.23450	-1.33300	C	1.78050	0.00000	0.00000

H	-0.62020	-1.39470	-1.33300
H	-1.51810	-0.16020	1.33300
H	1.47280	-2.02520	-0.13650
H	1.01750	2.28810	-0.13650
H	2.49030	0.26290	0.13660
H	-1.47280	2.02520	0.13650
H	-1.01750	-2.28810	0.13650

H	2.43370	-0.62410	-0.62410
H	2.43370	0.62410	0.62410
C	0.89250	0.89250	-0.89250
H	1.52610	1.52610	-1.52610

43

	x	y	z
H	-1.23650	1.99160	-0.29650
C	-0.75770	1.12680	0.18030
C	-0.58450	-1.20190	0.34300
C	1.55670	-0.19980	0.20820
C	0.58450	-1.20190	-0.34300
C	0.75770	1.12680	-0.18030
C	-1.55670	-0.19980	-0.20820
H	-0.50950	-1.16680	1.43540
H	1.65400	-0.26240	1.29800
H	0.50950	-1.16680	-1.43540
H	0.86460	1.26890	-1.26300
H	-1.65400	-0.26240	-1.29800
H	-0.86460	1.26890	1.26300
H	2.55800	-0.18170	-0.23830
H	1.23650	1.99160	0.29650
H	-2.55800	-0.18170	0.23830

44

	x	y	z
C	-1.52770	0.00000	0.00000
C	0.66270	1.22430	0.38670
C	0.66270	-1.22430	-0.38670
C	1.52770	0.00000	0.00000
C	-0.66270	-1.22440	0.38670
C	-0.66270	1.22440	-0.38660
H	-2.18540	0.25870	0.83840
H	-2.18520	-0.25880	-0.83850
H	-1.21830	2.15210	-0.20990
H	0.44220	-1.20030	-1.46200
H	1.21820	2.15210	0.21010
H	-0.44220	1.20040	-1.46200
H	0.44220	1.20030	1.46210
H	2.18540	-0.25880	0.83840
H	2.18530	0.25880	-0.83850
H	-1.21820	-2.15210	0.21000
H	-0.44220	-1.20030	1.46200
H	1.21830	-2.15210	-0.21000

45

	x	y	z
C	0.60990	-1.30160	-0.02270
C	-0.61000	-1.30160	0.02260
C	-1.58920	-0.19650	0.11740
H	-1.97310	-0.09810	1.14020
H	-2.45690	-0.28440	-0.54550
C	1.58920	-0.19660	-0.11730
H	1.97330	-0.09820	-1.14010
H	2.45680	-0.28450	0.54570
C	0.71790	1.05340	0.28750
H	1.23440	1.96580	-0.03580
H	0.66320	1.08530	1.38240
C	-0.71790	1.05340	-0.28750
H	-0.66310	1.08530	-1.38240
H	-1.23440	1.96580	0.03570

46

	x	y	z
C	0.00000	0.00000	0.00000
C	0.65170	-0.13260	-1.38840
H	1.68610	-0.48550	-1.30640
H	0.10320	-0.84500	-2.01520
H	0.66670	0.83100	-1.91060
C	0.79890	1.00650	0.84790
H	0.81780	1.99430	0.37340
H	0.35670	1.12140	1.84410
H	1.83620	0.67700	0.97750
C	-0.00250	-1.37180	0.69880
H	-0.46190	-1.30760	1.69190
H	-0.56520	-2.11010	0.11610
H	1.01780	-1.75160	0.82530
C	-1.44820	0.49790	-0.15830
H	-2.04190	-0.20070	-0.75900
H	-1.93820	0.60200	0.81660
H	-1.47730	1.47490	-0.65410

47

	x	y	z
C	0.15910	1.28140	0.13010
C	-1.13940	0.54020	-0.24700
C	-0.88970	-0.88850	0.25870
C	0.57020	-1.15230	-0.15440
C	1.28950	0.21630	0.01260
H	0.33990	2.16040	-0.49630
H	0.08710	1.63830	1.16410
H	-1.26350	0.52560	-1.33790
H	-2.03580	1.00410	0.17720
H	-0.98370	-0.91410	1.35250
H	-1.59100	-1.62480	-0.14750
H	1.04040	-1.95200	0.42640
H	0.59710	-1.46340	-1.20560
H	1.92280	0.22400	0.90540
H	1.94810	0.41960	-0.83770

48

	x	y	z
C	0.00000	0.00000	2.09380
C	0.00000	1.32200	1.29860
C	0.00000	-1.32200	-1.29860
C	-0.84730	1.38960	0.00000
C	0.00000	0.00000	-2.09380
C	0.00000	1.32200	-1.29860
H	-0.87340	-0.02100	2.75890
H	1.03450	1.57940	1.04100
H	-1.61390	0.60530	0.00000
H	-1.03450	-1.57940	-1.04100
H	-0.87340	-0.02100	-2.75890
H	0.87340	0.02100	2.75890
H	-0.32430	2.11120	1.98760
H	0.32430	-2.11120	-1.98760
H	-1.40010	2.33500	0.00000
H	0.87340	0.02100	-2.75890
H	-0.32430	2.11120	-1.98760

H	1.03450	1.57940	-1.04100
C	0.00000	-1.32200	1.29860
H	-1.03450	-1.57940	1.04100
H	0.32430	-2.11120	1.98760
C	0.84730	-1.38960	0.00000
H	1.61390	-0.60530	0.00000
H	1.40010	-2.33500	0.00000

49

	x	y	z
C	1.03040	0.33690	0.12590
C	0.33690	-1.03040	-0.12590
C	-0.33690	1.03040	-0.12600
C	-1.03040	-0.33690	0.12600
H	1.34800	0.44080	1.16880
H	1.86980	0.61140	-0.52000
H	0.44080	-1.34810	-1.16880
H	0.61140	-1.86980	0.52000
H	-0.61140	1.86990	0.51980
H	-0.44080	1.34800	-1.16900
H	-1.86990	-0.61140	-0.51990
H	-1.34800	-0.44070	1.16890

50

	x	y	z
C	0.00040	1.11610	-0.12680
C	-1.26970	0.19080	0.11270
C	1.26990	0.19010	0.11270
C	-0.61340	-1.15380	-0.08330
C	0.61250	-1.15400	-0.08330
H	0.00050	1.42850	-1.17660
H	0.00070	2.03160	0.47810
H	-2.08590	0.42230	-0.57790
H	-1.66510	0.28120	1.13090
H	1.66530	0.28020	1.13100
H	2.08630	0.42100	-0.57790

51

	x	y	z
C	-0.33230	-0.03290	-0.00060
C	1.05080	2.36240	-0.00070
C	-0.19350	2.28090	-0.00110
C	-0.88380	1.08550	-0.00120
C	2.00500	1.24230	0.00020
H	2.64100	1.21450	-0.88950
H	2.63960	1.21490	0.89100
C	1.11470	-0.29940	-0.00020
H	1.45600	-0.83600	0.89010
H	1.45640	-0.83580	-0.89050

52

	x	y	z
C	0.00000	0.00000	0.60560
C	0.00000	0.00000	-0.60560
H	0.00000	0.00000	-1.67050
H	0.00000	0.00000	1.67050

Table S3. The XYZ coordinates of the molecules presented in Fig. 3 and Table 2. The calculations were performed at the B3LYP/6-31G** level with the Grimmes D3 empirical dispersion correction.

1				2				3				4			
	x	y	z		x	y	z		x	y	z		x	y	z
C	0.7140	1.2367	-0.0435	C	0.4448	-1.3519	0.0076	C	0.4538	-1.3477	-0.0031	C	0.0121	1.4613	-0.0243
C	-0.7064	-1.2276	0.0145	C	-0.3898	1.3791	-0.0115	C	-0.3929	1.3684	0.0178	C	0.0273	-1.4482	-0.0387
C	1.4262	0.0086	-0.0543	C	1.3892	-0.3520	-0.0061	C	1.3816	-0.3439	0.0096	C	0.0321	0.7164	1.2476
C	-0.7149	1.2363	0.0288	C	-0.9994	-1.0271	0.0128	C	-0.9885	-1.0245	-0.0061	C	0.0040	0.7480	-1.2700
C	-1.4256	0.0073	0.0527	C	-1.3930	0.2908	0.0036	C	-1.3940	0.2810	0.0040	C	0.0089	-0.7261	-1.2792
C	0.7091	-1.2270	-0.0023	C	0.9484	1.0610	-0.0160	C	0.9404	1.0667	0.0205	C	0.0427	-0.7190	1.2424
C	-1.3613	2.5394	0.0154	C	-1.6914	-2.3083	0.0276	C	-1.6756	-2.2965	-0.0199	C	-0.0096	1.4709	-2.4760
C	1.3588	2.5409	-0.0442	C	-0.6698	-3.3586	0.0308	C	-0.6464	-3.3662	-0.0253	C	-0.0175	2.8566	-2.4463
C	1.4998	-2.4455	-0.1116	C	0.5859	-2.8103	0.0192	C	0.5973	-2.7952	-0.0157	C	-0.0119	3.5840	-1.2258
C	2.8771	-0.0868	-0.0905	C	-1.3270	-4.6506	0.0455	C	-1.3186	-4.6331	-0.0394	C	0.0032	2.8667	-0.0329
C	-2.8761	-0.0896	0.0913	C	-2.6762	-4.3765	0.0510	C	-2.6669	-4.3334	-0.0423	C	-0.0063	-1.4363	-2.4919
C	-1.4961	-2.4454	0.1391	C	2.1409	1.9123	-0.0300	C	2.1223	1.9147	0.0329	C	-0.0031	-2.8282	-2.5166
H	-2.3604	2.5969	-0.4162	C	3.2437	1.0991	-0.0289	C	3.2386	1.1231	0.0300	C	0.0149	-3.5214	-1.2765
H	2.3575	2.6040	0.3874	C	2.8449	-0.3107	-0.0144	C	2.8268	-0.3030	0.0156	C	0.0292	-2.8541	-0.0617
H	1.0256	-3.2858	-0.6201	C	4.0042	-1.0530	-0.0142	C	3.9512	-1.0725	0.0136	C	0.0673	-1.4142	2.4635
H	3.4463	0.7409	0.3307	C	5.1285	-0.1297	-0.0284	C	5.0866	-0.1431	0.0264	C	0.0790	-0.7396	3.6812
H	-3.4478	0.7347	-0.3327	C	4.6912	1.1759	-0.0374	C	4.6720	1.1744	0.0362	C	0.0658	0.6809	3.6617
H	-1.0225	-3.2765	0.6634	C	-2.7267	0.8978	0.0059	C	-2.7194	0.8806	0.0042	C	0.0435	1.3994	2.4766
B	2.8981	-2.4835	0.2527	C	-2.5739	2.2594	-0.0070	C	-2.5920	2.2430	0.0167	C	-0.0232	4.9790	-1.5733
B	3.5167	-1.3087	-0.5204	C	-1.1535	2.6190	-0.0181	C	-1.1511	2.5995	0.0257	C	-0.0156	-3.8267	-3.5508
B	0.6432	3.7340	-0.4369	C	-1.0905	3.9941	-0.0309	C	-1.0469	3.9579	0.0386	C	0.1029	-1.1361	5.0629
B	-0.6469	3.7377	0.3948	C	-2.4524	4.5060	-0.0280	C	-2.4195	4.4765	0.0380	H	-0.0138	0.9648	-3.4324
B	-3.5091	-1.3115	0.5305	H	4.1200	-2.1260	-0.0053	H	1.5517	-3.3085	-0.0166	H	0.0084	3.4249	0.8950
B	-2.8932	-2.4964	-0.2289	H	-0.2192	4.6310	-0.0416	H	-0.8732	-5.6188	-0.0461	H	-0.0223	-0.9118	-3.4391
				C	-3.3643	3.4745	-0.0137	H	2.0896	2.9978	0.0425	H	0.0402	-3.4293	0.8548
				C	-2.9138	-2.9412	0.0401	H	4.0556	-2.1490	0.0046	H	0.0786	-2.4969	2.4829
				H	-3.9010	-2.5049	0.0421	H	5.3030	2.0529	0.0461	H	0.0360	2.4807	2.5165
				H	6.1688	-0.4367	-0.0313	H	-3.6411	0.3107	-0.0045	H	-0.0227	5.8208	-0.8961
				H	-2.7068	5.5603	-0.0361	H	-0.1668	4.5865	0.0479	H	-0.0303	-3.6613	-4.6184
				H	-3.4622	-5.1239	0.0618	C	-3.3532	3.4587	0.0248	H	0.1177	-2.1435	5.4533
				C	2.1350	3.4027	-0.0432	H	-4.4296	3.5659	0.0212	C	0.1031	0.0157	5.8035
				H	1.6012	3.7754	-0.9241	C	-2.9042	-2.8855	-0.0303	H	0.1174	0.1509	6.8762
				H	1.6112	3.7911	0.8370	H	-3.8887	-2.4377	-0.0303	C	-0.0050	-5.0439	-2.9235
				H	3.1476	3.8074	-0.0526	O	6.3443	-0.6373	0.0270	H	-0.0090	-6.0406	-3.3428
				C	5.5373	2.4105	-0.0528	H	6.9730	0.0975	0.0361	C	-0.0347	5.0443	-2.9410
				H	5.3400	3.0272	-0.9387	O	-2.6204	5.8128	0.0494	H	-0.0452	5.9058	-3.5945
				H	5.3493	3.0428	0.8241	H	-3.5711	5.9899	0.0473	N	0.0806	1.1247	4.9711
				H	6.6005	2.1542	-0.0562	O	-3.7238	-5.1756	-0.0541	N	-0.0313	3.7687	-3.4852
				C	1.8795	-3.5506	0.0181	H	-3.4017	-6.0874	-0.0610	N	0.0135	-4.8773	-1.5469
				H	1.7235	-4.6298	0.0279					C	0.0750	2.5118	5.3787
				H	2.4686	-3.2903	-0.8679					H	0.9553	3.0380	4.9914
				H	2.4783	-3.2758	0.8933					H	-0.8230	3.0238	5.0138

				C	-0.6807	-6.0007	0.0532					H	0.0882	2.5678	6.4686
				H	-0.0484	-6.1534	-0.8305					C	0.0276	-5.9237	-0.5493
				H	-0.0386	-6.1387	0.9323					H	0.9236	-5.8544	0.0785
				H	-1.4342	-6.7933	0.0641					H	-0.8548	-5.8603	0.0981
				C	-4.0145	0.1476	0.0196					H	0.0254	-6.8957	-1.0457
				H	-4.0749	-0.4925	0.9066					C	-0.0407	3.4280	-4.8903
				H	-4.8713	0.8221	0.0179					H	0.8504	2.8489	-5.1592
				H	-4.0842	-0.5088	-0.8546					H	-0.9279	2.8364	-5.1443
				C	-4.8566	3.5898	-0.0064					H	-0.0521	4.3439	-5.4838
				H	-5.3060	3.1030	-0.8812								
				H	-5.2962	3.1182	0.8815								
				H	-5.1665	4.6386	-0.0138								
5				6				7				8			
	x	y	z		x	y	z		x	y	z		x	y	z
C	0.0147	1.4599	-0.0107	C	0.4604	-1.3475	0.0011	C	0.0148	1.4599	-0.0089	C	0.0148	1.4602	-0.0072
C	0.0240	-1.4501	-0.0346	C	-0.3989	1.3673	0.0035	C	0.0240	-1.4515	-0.0353	C	0.0240	-1.4514	-0.0373
C	0.0354	0.7107	1.2592	C	1.3836	-0.3380	0.0040	C	0.0354	0.7106	1.2609	C	0.0354	0.7090	1.2617
C	-0.0009	0.7507	-1.2588	C	-0.9845	-1.0290	-0.0006	C	-0.0010	0.7521	-1.2594	C	-0.0009	0.7538	-1.2585
C	0.0037	-0.7238	-1.2727	C	-1.3972	0.2752	0.0007	C	0.0036	-0.7224	-1.2735	C	0.0036	-0.7208	-1.2747
C	0.0401	-0.7249	1.2492	C	0.9370	1.0726	0.0051	C	0.0401	-0.7264	1.2485	C	0.0401	-0.7280	1.2476
C	-0.0204	1.4770	-2.4619	C	-1.6657	-2.3019	-0.0047	C	-0.0205	1.4754	-2.4637	C	-0.0204	1.4777	-2.4621
C	-0.0243	2.8622	-2.4289	C	-0.6338	-3.3718	-0.0038	C	-0.0246	2.8624	-2.4364	C	-0.0244	2.8641	-2.4341
C	-0.0091	3.5856	-1.2066	C	0.6095	-2.7918	-0.0011	C	-0.0092	3.5736	-1.2087	C	-0.0091	3.5737	-1.2044
C	0.0102	2.8644	-0.0153	C	-1.3018	-4.6332	-0.0141	C	0.0103	2.8632	-0.0133	C	0.0103	2.8628	-0.0087
C	-0.0117	-1.4301	-2.4866	C	-2.6620	-4.3511	-0.0191	C	-0.0117	-1.4279	-2.4864	C	-0.0118	-1.4235	-2.4884
C	-0.0075	-2.8225	-2.5155	N	-3.7002	-5.2496	0.0195	C	-0.0073	-2.8185	-2.5039	C	-0.0074	-2.8146	-2.5062
C	0.0128	-3.5194	-1.2779	C	2.1132	1.9238	0.0071	C	0.0130	-3.5262	-1.2742	C	0.0129	-3.5248	-1.2770
C	0.0282	-2.8553	-0.0620	C	3.2372	1.1370	0.0081	C	0.0284	-2.8562	-0.0595	C	0.0283	-2.8558	-0.0624
C	0.0600	-1.4231	2.4677	C	2.8266	-0.2915	0.0074	C	0.0600	-1.4242	2.4659	C	0.0600	-1.4280	2.4629
C	0.0752	-0.7519	3.6879	C	3.9536	-1.0563	0.0118	C	0.0750	-0.7440	3.6788	C	0.0750	-0.7479	3.6764
C	0.0700	0.6684	3.6727	C	5.0993	-0.1299	0.0194	C	0.0701	0.6749	3.6768	C	0.0701	0.6719	3.6769
C	0.0506	1.3896	2.4897	C	4.6636	1.1892	0.0169	C	0.0507	1.3920	2.4894	C	0.0506	1.3894	2.4905
C	-0.0184	4.9840	-1.5451	N	6.3964	-0.5797	-0.0216	C	-0.0184	4.9695	-1.5431	C	-0.0185	4.9583	-1.5582
C	-0.0193	-3.8149	-3.5571	C	-2.7225	0.8682	0.0004	C	-0.0192	-3.8061	-3.5455	C	-0.0190	-3.8134	-3.5284
C	0.0963	-1.1579	5.0680	C	-2.6032	2.2350	0.0025	C	0.0960	-1.1523	5.0547	C	0.0960	-1.1338	5.0524
H	-0.0324	0.9747	-3.4210	C	-1.1607	2.5938	0.0057	H	-0.0326	0.9705	-3.4213	H	-0.0323	0.9745	-3.4205
H	0.0218	3.4206	0.9137	C	-1.0621	3.9522	0.0112	H	0.0218	3.4295	0.9086	H	0.0218	3.4276	0.9140
H	-0.0272	-0.9036	-3.4326	C	-2.4372	4.4811	0.0161	H	-0.0273	-0.9126	-3.4377	H	-0.0275	-0.9068	-3.4389
H	0.0434	-3.4347	0.8525	N	-2.6963	5.8293	-0.0245	H	0.0437	-3.4331	0.8565	H	0.0436	-3.4342	0.8525
H	0.0639	-2.5058	2.4847	H	1.5662	-3.3009	0.0003	H	0.0640	-2.5058	2.4952	H	0.0641	-2.5096	2.4905
H	0.0476	2.4713	2.5343	H	-0.8538	-5.6175	-0.0184	H	0.0476	2.4738	2.5311	H	0.0475	2.4711	2.5340
H	-0.0113	5.8193	-0.8599	H	-4.5979	-4.9306	-0.3133	C	0.1030	-0.0200	5.8277	C	0.1032	-0.0123	5.8325
H	-0.0354	-3.6392	-4.6231	H	-3.4896	-6.2075	-0.2191	H	0.1177	0.0909	6.9021	H	0.1181	0.0862	6.9064
H	0.1052	-2.1690	5.4488	H	2.0759	3.0069	0.0053	C	-0.0064	-5.0416	-2.9514	C	-0.0064	-5.0497	-2.9473
C	0.1031	-0.0142	5.8200	H	4.0425	-2.1360	0.0029	H	-0.0096	-6.0277	-3.3926	H	-0.0098	-6.0291	-3.3989
H	0.1180	0.1102	6.8933	H	5.2920	2.0693	0.0209	C	-0.0385	5.0727	-2.9100	C	-0.0384	5.0731	-2.9194

C	-0.0063	-5.0380	-2.9427	H	6.5697	-1.5174	0.3087	H	-0.0504	5.9478	-3.5432	H	-0.0502	5.9539	-3.5417
H	-0.0093	-6.0299	-3.3716	H	7.1214	0.0812	0.2158	N	0.0876	1.0965	4.9870	N	0.0874	1.0948	4.9898
C	-0.0382	5.0634	-2.9114	H	-3.6418	0.2943	-0.0024	H	0.0893	2.0534	5.2948	H	0.0886	2.0530	5.2954
H	-0.0502	5.9307	-3.5558	H	-0.1715	4.5691	0.0049	N	-0.0421	3.7863	-3.4566	N	-0.0419	3.7897	-3.4567
N	0.0874	1.0930	4.9872	H	-1.9716	6.4481	0.3076	H	-0.0553	3.5743	-4.4391	H	-0.0551	3.5751	-4.4393
H	0.0885	2.0521	5.2910	H	-3.6317	6.1264	0.2114	N	0.0134	-4.8717	-1.5642	N	0.0132	-4.8734	-1.5672
N	-0.0419	3.7884	-3.4538	C	-3.3617	3.4442	0.0106	H	0.0259	-5.6166	-0.8894	H	0.0256	-5.6170	-0.8902
H	-0.0553	3.5719	-4.4362	H	-4.4381	3.5483	0.0125	O	-0.0397	-3.4688	-4.8717	F	-0.0392	-3.5607	-4.8491
N	0.0131	-4.8702	-1.5675	C	-2.8915	-2.8957	-0.0112	H	-0.0448	-4.2759	-5.4013	F	0.1067	-2.4041	5.4938
H	0.0254	-5.6128	-0.8889	H	-3.8710	-2.4329	-0.0043	O	0.1057	-2.4696	5.4257	F	-0.0092	5.9760	-0.6791
								H	0.1195	-2.5247	6.3894				
								O	-0.0078	5.9495	-0.5878				
								H	-0.0168	6.8117	-1.0218				

	9				10		
	x	y	z		x	y	z
C	0.4535	-1.3450	0.0010	C	0.4523	-1.3501	0.0009
C	-0.3988	1.3815	-0.0064	C	-0.3944	1.3781	-0.0062
C	1.3958	-0.3454	-0.0047	C	1.3907	-0.3476	-0.0047
C	-0.9971	-1.0361	0.0034	C	-0.9962	-1.0306	0.0031
C	-1.3915	0.2798	0.0002	C	-1.3952	0.2833	-0.0002
C	0.9381	1.0652	-0.0091	C	0.9431	1.0666	-0.0086
C	-1.6880	-2.3169	0.0091	C	-1.6843	-2.3138	0.0087
C	-0.6718	-3.3740	0.0104	C	-0.6566	-3.3574	0.0099
C	0.5644	-2.8015	0.0052	C	0.5982	-2.8065	0.0052
C	-1.3244	-4.6532	0.0152	C	-1.3057	-4.6410	0.0149
C	-2.6728	-4.3883	0.0160	C	-2.6579	-4.3885	0.0164
C	2.1440	1.8895	-0.0156	C	2.1314	1.9212	-0.0147
C	3.2579	1.1051	-0.0156	C	3.2359	1.1100	-0.0147
C	2.8505	-0.3034	-0.0084	C	2.8460	-0.3019	-0.0085
C	4.0080	-1.0456	-0.0068	C	4.0091	-1.0391	-0.0082
C	5.1368	-0.1207	-0.0134	C	5.1296	-0.1076	-0.0142
C	4.6920	1.1795	-0.0190	C	4.6721	1.1897	-0.0182
C	-2.7084	0.9120	0.0023	C	-2.7295	0.8851	0.0014
C	-2.5861	2.2688	-0.0030	C	-2.5794	2.2472	-0.0036
C	-1.1626	2.6203	-0.0082	C	-1.1617	2.6155	-0.0083
C	-1.0987	3.9938	-0.0113	C	-1.1049	3.9914	-0.0121
C	-2.4641	4.5088	-0.0088	C	-2.4719	4.4960	-0.0101
H	4.1090	-2.1190	-0.0015	H	4.1295	-2.1117	-0.0042
H	-0.2197	4.6181	-0.0148	H	-0.2363	4.6321	-0.0160
C	-3.3676	3.4735	-0.0041	C	-3.3665	3.4511	-0.0050
C	-2.9094	-2.9483	0.0122	C	-2.9043	-2.9524	0.0126
H	-3.8895	-2.4992	0.0118	H	-3.8935	-2.5203	0.0129
H	6.1739	-0.4345	-0.0137	H	6.1696	-0.4130	-0.0152
H	-2.7110	5.5639	-0.0105	H	-2.7275	5.5493	-0.0124
H	-3.4630	-5.1296	0.0190	H	-3.4424	-5.1365	0.0200

H	-0.8425	-5.6208	0.0171	H	-0.8267	-5.6112	0.0170	
H	-4.4466	3.5400	-0.0011	H	-4.4462	3.5212	-0.0026	
H	5.2891	2.0806	-0.0243	H	5.2728	2.0896	-0.0230	
Cl	2.1948	3.6159	-0.0235	C	2.1419	3.4107	-0.0201	
Cl	-4.2289	0.0928	0.0105	H	1.6178	3.7971	-0.9009	
Cl	2.0342	-3.7086	0.0038	H	1.6237	3.8035	0.8614	
				H	3.1640	3.7956	-0.0249	
				C	1.8829	-3.5603	0.0045	
				H	2.4793	-3.3058	-0.8783	
				H	2.4825	-3.3017	0.8840	
				H	1.7053	-4.6379	0.0074	
				C	-4.0247	0.1493	0.0073	
				H	-4.1040	-0.4993	-0.8718	
				H	-4.0987	-0.4945	0.8905	
				H	-4.8690	0.8419	0.0079	

Table S4. The XYZ coordinates of the hexacene molecules presented in Fig. 4. The calculations were performed at the B3LYP/6-31G** level with the Grimmes D3 empirical dispersion correction.

0000				0001				0002				0010			
	x	y	z		x	y	z		x	y	z		x	y	z
C	0.0000	0.0000	1.4448	C	3.0225	1.5698	0.4909	C	5.4847	-1.4419	-0.2802	C	-3.4818	-2.2076	0.3647
C	0.0000	0.0000	2.8729	C	3.4759	0.3242	0.0317	C	4.2718	-1.8840	-0.7670	C	-4.4873	-1.3055	0.2362
C	-0.1325	1.2871	0.7842	C	1.6710	1.9175	0.4706	C	5.5571	-0.1996	0.3799	C	-2.1061	-1.8070	0.2439
C	0.1325	-1.2871	0.7842	C	4.8523	-0.0500	0.0392	C	4.4262	0.5801	0.5325	C	-4.2187	0.0846	-0.0257
C	-0.7181	1.4825	-0.5357	C	2.5098	-0.5933	-0.5108	C	3.0987	-1.1084	-0.6125	C	-1.0698	-2.7400	0.3103
C	0.2384	2.4512	1.5097	C	0.6834	0.9546	0.0387	C	3.1659	0.1566	0.0434	C	-1.7847	-0.4313	-0.0048
C	0.2459	1.2090	3.5855	C	1.2562	3.2575	0.7778	C	1.8415	-1.5550	-1.1241	C	-5.2791	1.0074	-0.1584
C	-0.2459	-1.2090	3.5855	C	2.9651	-1.8402	-1.0342	C	0.6995	-0.8441	-0.9148	C	-2.8717	0.5358	-0.1490
C	-0.2384	-2.4512	1.5097	C	5.2518	-1.2621	-0.4649	C	1.9552	0.9506	0.1812	C	0.2756	-2.3740	0.1877
C	0.7181	-1.4825	-0.5357	C	1.1489	-0.2457	-0.5016	C	0.7007	0.3985	-0.1952	C	-0.4371	-0.0714	-0.1022
C	-1.4875	0.4939	-1.1988	C	-0.7364	1.3230	0.0638	C	2.0026	2.3069	0.6155	C	-2.6536	1.9095	-0.3947
C	-0.5914	2.7544	-1.1763	C	-0.0241	3.6461	0.5532	C	-0.5208	1.1528	0.0186	C	-5.0347	2.3457	-0.4053
C	0.3415	3.7132	0.8414	C	4.2973	-2.1676	-1.0091	C	0.8843	3.0914	0.5809	C	1.3019	-3.3690	0.1061
C	0.4615	2.3780	2.9152	C	-1.8368	0.3969	-0.0843	C	-0.3892	2.5516	0.2490	C	0.6211	-0.9825	0.0567
C	-0.4615	-2.3780	2.9152	C	-1.0366	2.7078	0.1473	C	-1.8629	0.6058	-0.0692	C	-3.7091	2.7968	-0.5229
C	-0.3415	-3.7132	0.8414	C	-1.8131	-1.0118	0.3031	C	-1.5147	3.4127	0.0957	C	2.0289	-0.6048	-0.0067
C	1.4875	-0.4939	-1.1988	C	-2.3339	3.1803	-0.1868	C	-2.9253	1.4999	-0.3722	C	2.5837	-3.0106	-0.1686
C	0.5914	-2.7544	-1.1763	C	-3.0743	0.9090	-0.5709	C	-2.2228	-0.7748	0.2263	C	2.5266	0.7501	0.1583
C	-1.1136	2.9406	-2.4794	C	-0.8637	-1.5531	1.2080	C	-2.7186	2.9093	-0.3016	C	2.9736	-1.6318	-0.2464
C	-2.0151	0.7096	-2.4558	C	-4.1135	0.0130	-0.9818	C	-4.2202	0.9918	-0.7066	C	1.7857	1.8027	0.7632
C	0.0000	3.8478	-0.4693	C	-2.8627	-1.8776	-0.1357	C	-1.3891	-1.6537	0.9669	C	3.8801	1.0447	-0.2147
C	2.0151	-0.7096	-2.4558	C	-3.2938	2.3116	-0.6277	C	-3.5242	-1.2522	-0.1291	C	4.3283	-1.3082	-0.5612
C	0.0000	-3.8478	-0.4693	C	-2.8311	-3.2520	0.2065	C	-1.7654	-2.9541	1.2354	C	4.3651	2.3727	-0.1217
C	1.1136	-2.9406	-2.4794	C	-3.9846	-1.3335	-0.8374	C	-4.4917	-0.3417	-0.6551	C	4.7515	-0.0134	-0.6097
C	-1.8027	1.9332	-3.1207	C	-0.8743	-2.8885	1.5583	C	-3.8712	-2.6024	0.1239	C	2.2948	3.0816	0.8738
C	1.8027	-1.9332	-3.1207	C	-1.8464	-3.7596	1.0269	C	-3.0048	-3.4491	0.7815	C	3.5840	3.3847	0.3926
H	0.2971	1.1708	4.6701	H	3.7486	2.3033	0.8339	H	6.3780	-2.0475	-0.4002	H	-3.6982	-3.2550	0.5569
H	-0.2971	-1.1708	4.6701	H	5.5795	0.6456	0.4500	H	4.1984	-2.8418	-1.2757	H	-5.5239	-1.6190	0.3274
H	-1.6794	-0.4482	-0.7033	H	2.0041	3.9691	1.1167	H	6.5070	0.1482	0.7751	H	-1.3163	-3.7930	0.4259
H	0.7027	4.5660	1.4099	H	2.2321	-2.5307	-1.4430	H	4.5147	1.5234	1.0590	H	-6.2995	0.6455	-0.0618
H	0.7258	3.2862	3.4497	H	6.3023	-1.5373	-0.4534	H	1.8097	-2.4797	-1.6940	H	-0.1938	0.9471	-0.3613
H	-0.7258	-3.2862	3.4497	H	0.4454	-0.9485	-0.9297	H	-0.2326	-1.2055	-1.3279	H	-1.6425	2.2910	-0.4851
H	1.6794	0.4482	-0.7033	H	-0.3193	4.6825	0.6929	H	2.9495	2.7547	0.8916	H	-5.8601	3.0442	-0.5055
H	-0.7027	-4.5660	1.4099	H	4.6317	-3.1224	-1.4039	H	0.9530	4.1519	0.8075	H	1.0241	-4.4144	0.2077
H	-0.9840	3.9090	-2.9558	H	-2.5287	4.2482	-0.1426	H	-1.3775	4.4804	0.2425	H	-3.5083	3.8468	-0.7138
H	-2.6024	-0.0707	-2.9304	H	-5.0111	0.4389	-1.4219	H	-3.5572	3.5688	-0.5067	H	3.3512	-3.7676	-0.3052
H	0.1051	4.8031	-0.9763	H	-4.2568	2.6761	-0.9744	H	-0.4438	-1.2904	1.3475	H	5.0129	-2.1212	-0.7873
H	2.6024	0.0707	-2.9304	H	-0.1187	-0.9019	1.6449	H	-4.9865	1.7006	-1.0086	H	0.8203	1.5906	1.2023
H	0.9840	-3.9090	-2.9558	H	-4.7657	-2.0075	-1.1781	H	-1.1041	-3.5944	1.8114	H	5.7726	0.2276	-0.8923
H	-0.1051	-4.8031	-0.9763	H	-3.6240	-3.8960	-0.1650	H	-4.8545	-2.9501	-0.1823	H	5.3851	2.5730	-0.4393

H	-2.2051	2.0907	-4.1169	H	-0.1331	-3.2640	2.2573	H	-5.4698	-0.7217	-0.9370	H	1.7015	3.8531	1.3558
H	2.2051	-2.0907	-4.1169	H	-1.8397	-4.8128	1.2912	H	-3.2878	-4.4784	0.9806	H	3.9717	4.3962	0.4664
0011				0012				0020				0021			
	x	y	z		x	y	z		x	y	z		x	y	z
C	1.0701	2.2817	0.3462	C	3.6395	0.1449	0.0668	C	-6.3342	-1.0888	-0.0057	C	3.1274	-1.6624	-0.4139
C	1.9985	1.2276	0.2023	C	4.2118	-1.1271	-0.2272	C	-5.8444	0.1773	0.2369	C	4.2605	-0.8872	-0.1242
C	-0.2985	2.0818	0.2349	C	4.5105	1.2025	0.4074	C	-5.4381	-2.1362	-0.2991	C	1.8418	-1.1230	-0.3921
C	3.3880	1.4139	0.3093	C	2.1879	0.2981	0.0096	C	-4.0776	-1.9018	-0.3500	C	5.5832	-1.4214	-0.1349
C	1.4854	-0.0845	-0.1235	C	5.6140	-1.2869	-0.1845	C	-4.4546	0.4416	0.1898	C	4.0743	0.5014	0.2036
C	-0.8315	0.7412	0.0345	C	3.3561	-2.2364	-0.5588	C	-3.5413	-0.6111	-0.1160	C	1.6471	0.2708	-0.0812
C	-1.1980	3.2027	0.2181	C	5.8833	1.0232	0.4446	C	-3.9422	1.7473	0.4565	C	0.6920	-1.9203	-0.7046
C	2.3938	-1.1346	-0.3462	C	1.5334	1.5187	0.1797	C	-2.6050	2.0076	0.4153	C	5.2217	1.2915	0.5118
C	4.2850	0.3655	0.1014	C	1.3747	-0.8452	-0.2891	C	-2.1191	-0.3221	-0.1660	C	6.6627	-0.6291	0.1639
C	0.0849	-0.2772	-0.2039	C	6.4433	-0.2301	0.1437	C	-1.6475	0.9885	0.1010	C	2.7747	1.0385	0.2123
C	-2.2859	0.5594	-0.0104	C	2.0056	-2.1038	-0.5841	C	-1.1612	-1.3140	-0.5171	C	0.2901	0.8155	-0.0760
C	-2.5138	3.0263	-0.0531	C	0.1372	1.6425	0.1308	C	-0.2279	1.2739	0.0513	C	-0.5669	-1.4197	-0.6058
C	5.7024	0.5394	0.2084	C	-0.0198	-0.7297	-0.3037	C	0.1828	-1.0641	-0.4847	C	6.4796	0.7438	0.4919
C	3.7727	-0.9472	-0.2403	C	-0.4721	2.9379	0.1581	C	0.7095	0.2130	-0.1242	C	-0.8255	-0.0553	-0.2104
C	-3.0860	1.7105	-0.1867	C	-0.6888	0.4738	-0.0311	C	0.2654	2.6107	0.1094	C	0.0599	2.2136	0.0089
C	-2.9562	-0.7205	0.1115	C	-2.1404	0.6211	-0.0126	C	1.5959	2.8766	-0.0449	C	-1.2074	2.7251	-0.0770
C	4.7066	-2.0112	-0.4556	C	-1.8099	3.0729	-0.0387	C	2.1327	0.4803	-0.0566	C	-2.1661	0.4650	-0.0741
C	6.5584	-0.5059	-0.0060	C	-3.0786	-0.4789	0.1282	C	2.5562	1.8310	-0.1365	C	-3.3571	-0.3560	0.1145
C	-4.3472	-0.8150	-0.2325	C	-2.6685	1.9283	-0.1428	C	3.1590	-0.5431	0.0883	C	-2.3411	1.8748	-0.1252
C	-2.3487	-1.8907	0.6466	C	-2.7290	-1.7635	0.6270	C	2.9057	-1.8598	0.5562	C	-3.3278	-1.6991	0.5724
C	-4.4760	1.5878	-0.4778	C	-4.4665	-0.2515	-0.1549	C	3.9386	2.1497	-0.3218	C	-4.6476	0.2395	-0.0623
C	6.0535	-1.7997	-0.3430	C	-4.0643	2.1250	-0.3717	C	4.5282	-0.1928	-0.1490	C	-3.6512	2.4397	-0.2490
C	-5.0050	-2.0697	-0.1784	C	-5.3877	-1.3253	-0.0812	C	3.9115	-2.8021	0.6605	C	-5.8131	-0.5523	0.0649
C	-5.0745	0.3641	-0.5623	C	-4.9235	1.0693	-0.4406	C	4.8814	1.1712	-0.3922	C	-4.7568	1.6492	-0.2848
C	-3.0237	-3.0918	0.7221	C	-3.6540	-2.7844	0.7218	C	5.5348	-1.1845	-0.0656	C	-4.4833	-2.4445	0.7199
C	-4.3555	-3.1978	0.2705	C	-4.9917	-2.5795	0.3296	C	5.2360	-2.4767	0.3126	C	-5.7391	-1.8809	0.4299
H	1.4440	3.2907	0.5038	H	4.1054	2.1769	0.6568	H	-7.4022	-1.2810	0.0326	H	3.2536	-2.7149	-0.6572
H	3.7709	2.4017	0.5553	H	6.0345	-2.2627	-0.4134	H	-6.5216	0.9946	0.4708	H	5.7187	-2.4706	-0.3841
H	-0.7875	4.1977	0.3666	H	6.5279	1.8555	0.7111	H	-5.8184	-3.1366	-0.4836	H	0.8471	-2.9416	-1.0421
H	2.0095	-2.1205	-0.5976	H	3.8202	-3.1933	-0.7824	H	-3.4169	-2.7330	-0.5669	H	5.0803	2.3399	0.7616
H	-0.2610	-1.2581	-0.5027	H	2.1070	2.4297	0.3144	H	-4.6438	2.5389	0.7061	H	7.6652	-1.0465	0.1527
H	-3.1794	3.8811	-0.1370	H	7.5200	-0.3677	0.1734	H	-2.2667	3.0089	0.6516	H	2.6713	2.0840	0.4820
H	6.0846	1.5241	0.4646	H	1.3715	-2.9527	-0.8258	H	-1.4924	-2.2932	-0.8415	H	-1.4004	-2.0408	-0.9045
H	7.6311	-0.3592	0.0786	H	-0.5868	-1.6061	-0.5889	H	0.8608	-1.8411	-0.8107	H	7.3447	1.3568	0.7266
H	4.3203	-2.9943	-0.7115	H	0.1636	3.8106	0.2804	H	-0.4229	3.4408	0.2060	H	0.8978	2.8975	0.0712
H	-5.0482	2.4945	-0.6543	H	-2.2633	4.0590	-0.0908	H	1.9456	3.9049	-0.0792	H	-1.3621	3.8005	-0.0945
H	-1.3506	-1.8299	1.0583	H	-4.4246	3.1401	-0.5146	H	1.9116	-2.1244	0.8905	H	-3.7384	3.5190	-0.3400
H	6.7502	-2.6159	-0.5094	H	-1.7274	-1.9365	0.9959	H	4.2155	3.1948	-0.4303	H	-2.3851	-2.1428	0.8638
H	-6.1248	0.2759	-0.8262	H	-5.9772	1.2230	-0.6567	H	3.6774	-3.7950	1.0332	H	-5.7452	2.0804	-0.4185
H	-6.0502	-2.1171	-0.4732	H	-6.4280	-1.1308	-0.3290	H	6.5616	-0.8978	-0.2775	H	-6.7786	-0.0817	-0.1014
H	-2.5285	-3.9573	1.1524	H	-3.3488	-3.7469	1.1217	H	5.9246	1.4155	-0.5728	H	-4.4178	-3.4664	1.0817
H	-4.8738	-4.1508	0.3160	H	-5.7100	-3.3915	0.3904	H	6.0197	-3.2250	0.3813	H	-6.6427	-2.4742	0.5327

0022				0101				0102				0110			
	x	y	z		x	y	z		x	y	z		x	y	z
C	1.2266	5.6990	0.8568	C	2.7396	1.2857	0.0000	C	-3.1379	-1.7175	0.0000	C	0.0000	0.0000	0.0990
C	-0.0240	5.5998	0.2839	C	1.8804	2.3923	0.0000	C	-4.5489	-1.5121	0.0000	C	0.0000	0.0000	1.5391
C	1.9379	4.5237	1.1652	C	2.2646	-0.0253	0.0000	C	-2.6652	-3.0483	0.0000	C	0.0000	1.2350	-0.5868
C	1.4216	3.2842	0.8382	C	2.3974	3.7323	0.0000	C	-2.2507	-0.5539	0.0000	C	0.0000	-1.2350	-0.5868
C	-0.5873	4.3410	-0.0350	C	0.4653	2.1752	0.0000	C	-5.4175	-2.6252	0.0000	C	0.0000	2.4559	0.0755
C	0.1745	3.1456	0.1731	C	0.8501	-0.2572	0.0000	C	-5.0788	-0.1733	0.0000	C	0.0000	1.2394	2.2105
C	-1.9379	4.2515	-0.4957	C	3.1758	-1.1438	0.0000	C	-3.5350	-4.1260	0.0000	C	0.0000	-2.4559	0.0755
C	-2.5294	3.0379	-0.6674	C	-0.4280	3.3302	0.0000	C	-0.8567	-0.6624	0.0000	C	0.0000	-1.2394	2.2105
C	-0.4042	1.8639	-0.2017	C	1.5644	4.8048	0.0000	C	-2.8308	0.7600	0.0000	C	0.0000	3.7454	-0.6227
C	-1.7875	1.8250	-0.5087	C	0.0000	0.8509	0.0000	C	-4.9243	-3.9168	0.0000	C	0.0000	2.4473	1.5240
C	0.3455	0.6258	-0.2892	C	0.3558	-1.6405	0.0000	C	-4.2613	0.9091	0.0000	C	0.0000	-3.7454	-0.6227
C	-2.4275	0.5699	-0.7036	C	2.7246	-2.4202	0.0000	C	0.0000	0.4498	0.0000	C	0.0000	-2.4473	1.5240
C	-0.3455	-0.6258	-0.2892	C	0.1362	4.6411	0.0000	C	-1.9902	1.8738	0.0000	C	0.0000	4.9482	0.1404
C	1.7527	0.6064	-0.5376	C	1.3131	-2.7208	0.0000	C	1.4532	0.3344	0.0000	C	0.0000	3.8556	-2.0289
C	-1.7527	-0.6064	-0.5376	C	-0.9968	-1.9655	0.0000	C	-0.5958	1.7541	0.0000	C	0.0000	3.6994	2.2375
C	2.4275	-0.5699	-0.7036	C	-1.4555	-3.2980	0.0000	C	2.0998	-0.9404	0.0000	C	0.0000	-4.9482	0.1404
C	0.4042	-1.8639	-0.2017	C	0.8705	-4.0400	0.0000	C	0.2512	2.9059	0.0000	C	0.0000	-3.8556	-2.0289
C	-0.1745	-3.1456	0.1731	C	-2.8431	-3.6212	0.0000	C	2.2535	1.5030	0.0000	C	0.0000	-3.6994	2.2375
C	1.7875	-1.8250	-0.5087	C	-0.4971	-4.3682	0.0000	C	3.6998	1.3904	0.0000	C	0.0000	6.1944	-0.5198
C	-1.4216	-3.2842	0.8382	C	-3.2610	-4.9297	0.0000	C	3.4592	-1.0571	0.0000	C	0.0000	4.8848	1.5817
C	0.5873	-4.3410	-0.0350	C	-0.9660	-5.7133	0.0000	C	1.6064	2.7847	0.0000	C	0.0000	5.0904	-2.6591
C	2.5294	-3.0379	-0.6674	C	-2.3118	-5.9878	0.0000	C	4.5673	2.5135	0.0000	C	0.0000	-6.1944	-0.5198
C	0.0240	-5.5998	0.2839	H	3.8137	1.4567	0.0000	C	4.2996	0.0937	0.0000	C	0.0000	-4.8848	1.5817
C	-1.9379	-4.5237	1.1652	H	3.4754	3.8688	0.0000	C	5.7104	-0.0325	0.0000	C	0.0000	-5.0904	-2.6591
C	1.9379	-4.2515	-0.4957	H	4.2421	-0.9344	0.0000	C	5.9396	2.3650	0.0000	C	0.0000	6.2720	-1.9018
C	-1.2266	-5.6990	0.8568	H	-1.0682	0.6817	0.0000	C	6.5223	1.0807	0.0000	C	0.0000	-6.2720	-1.9018
H	1.6428	6.6710	1.1039	H	3.4232	-3.2526	0.0000	H	-1.5991	-3.2452	0.0000	H	0.0000	1.2002	-1.6712
H	-0.6129	6.4931	0.0929	H	-1.7484	-1.1830	0.0000	H	-6.4900	-2.4484	0.0000	H	0.0000	-1.2002	-1.6712
H	2.8909	4.5871	1.6821	H	1.6043	-4.8428	0.0000	H	-3.1384	-5.1369	0.0000	H	0.0000	1.2481	3.2980
H	1.9645	2.3990	1.1425	H	-3.5678	-2.8111	0.0000	H	-6.1584	-0.0490	0.0000	H	0.0000	-1.2481	3.2980
H	-2.4974	5.1697	-0.6519	H	-0.2372	-6.5196	0.0000	H	-0.4212	-1.6515	0.0000	H	0.0000	2.9616	-2.6423
H	-3.5756	2.9673	-0.9525	H	-4.3217	-5.1628	0.0000	H	-5.6042	-4.7634	0.0000	H	0.0000	3.6686	3.3237
H	2.2734	1.5400	-0.7029	H	-2.6581	-7.0171	0.0000	H	-4.6732	1.9146	0.0000	H	0.0000	-2.9616	-2.6423
H	-3.4773	0.5591	-0.9835	H	1.9661	5.8146	0.0000	H	-2.4315	2.8679	0.0000	H	0.0000	-3.6686	3.3237
H	-2.2734	-1.5400	-0.7029	C	-1.8367	3.2185	0.0000	H	1.5041	-1.8451	0.0000	H	0.0000	7.1014	0.0792
H	3.4773	-0.5591	-0.9835	C	-0.7152	5.7687	0.0000	H	-0.2110	3.8893	0.0000	H	0.0000	5.1402	-3.7439
H	-1.9645	-2.3990	1.1425	C	-2.0897	5.6257	0.0000	H	3.9226	-2.0401	0.0000	H	0.0000	5.8203	2.1348
H	3.5756	-2.9673	-0.9525	C	-2.6520	4.3372	0.0000	H	2.2066	3.6860	0.0000	H	0.0000	-5.8203	2.1348
H	0.6129	-6.4931	0.0929	H	-2.3026	2.2394	0.0000	H	4.1585	3.5169	0.0000	H	0.0000	-7.1014	0.0792
H	-2.8909	-4.5871	1.6821	H	-0.2668	6.7588	0.0000	H	6.1424	-1.0300	0.0000	H	0.0000	-5.1402	-3.7439
H	2.4974	-5.1697	-0.6519	H	-2.7318	6.5016	0.0000	H	6.5750	3.2457	0.0000	H	0.0000	7.2386	-2.3964
H	-1.6428	-6.6710	1.1039	H	-3.7310	4.2160	0.0000	H	7.6028	0.9733	0.0000	H	0.0000	-7.2386	-2.3964
0111				0112				0120				0202			
	x	y	z		x	y	z		x	y	z		x	y	z

C	1.8746	-3.4466	0.0000	C	3.7479	1.4385	0.0000	C	-3.6493	-0.4815	0.0000	C	0.0933	6.8256	0.0000
C	3.2608	-3.5559	0.0000	C	4.9493	0.6732	0.0000	C	-4.4568	-1.5719	0.0000	C	1.1973	5.9995	0.0000
C	1.2249	-2.1927	0.0000	C	3.8605	2.8444	0.0000	C	-2.2167	-0.6135	0.0000	C	-1.1973	6.2597	0.0000
C	3.9284	-4.8257	0.0000	C	2.4570	0.7425	0.0000	C	-3.9148	-2.9057	0.0000	C	-1.3622	4.8882	0.0000
C	4.0701	-2.3483	0.0000	C	6.1967	1.3314	0.0000	C	-1.3990	0.5213	0.0000	C	1.0530	4.5914	0.0000
C	2.0353	-0.9837	0.0000	C	4.8835	-0.7679	0.0000	C	-1.6286	-1.9240	0.0000	C	-0.2499	4.0098	0.0000
C	-0.1755	-2.0730	0.0000	C	2.4462	-0.7060	0.0000	C	-4.7702	-4.0290	0.0000	C	2.1943	3.7341	0.0000
C	5.4983	-2.4829	0.0000	C	1.2371	1.4062	0.0000	C	-2.5022	-3.0960	0.0000	C	2.0639	2.3777	0.0000
C	3.4419	-1.1078	0.0000	C	5.0964	3.4725	0.0000	C	0.0000	0.4447	0.0000	C	-0.3789	2.5625	0.0000
C	1.3929	0.2668	0.0000	C	3.6968	-1.4214	0.0000	C	-0.2357	-2.0031	0.0000	C	0.7789	1.7430	0.0000
C	-0.8076	-0.8264	0.0000	C	1.2371	-1.3912	0.0000	C	-2.0119	-4.4199	0.0000	C	-1.6508	1.9234	0.0000
C	5.2923	-4.9041	0.0000	C	0.0023	0.7200	0.0000	C	-4.2608	-5.3145	0.0000	C	0.6444	0.3005	0.0000
C	0.0000	0.3803	0.0000	C	6.2766	2.7131	0.0000	C	0.8700	1.6131	0.0000	C	-1.7782	0.5625	0.0000
C	-2.2182	-0.6834	0.0000	C	-0.0023	-0.7200	0.0000	C	0.5840	-0.8651	0.0000	C	-0.6444	-0.3005	0.0000
C	6.0895	-3.7145	0.0000	C	-1.2371	1.3912	0.0000	C	-2.8690	-5.5078	0.0000	C	1.7782	-0.5625	0.0000
C	-2.8336	0.5551	0.0000	C	-2.4462	0.7060	0.0000	C	0.3343	2.9379	0.0000	C	1.6508	-1.9234	0.0000
C	-0.6540	1.6421	0.0000	C	-1.2371	-1.4062	0.0000	C	2.0089	-0.9856	0.0000	C	-0.7789	-1.7430	0.0000
C	-4.2723	0.6633	0.0000	C	-3.6968	1.4214	0.0000	C	2.2757	1.4451	0.0000	C	0.3789	-2.5625	0.0000
C	-2.0300	1.7693	0.0000	C	-2.4570	-0.7425	0.0000	C	3.1429	2.6077	0.0000	C	-2.0639	-2.3777	0.0000
C	-4.8899	1.8676	0.0000	C	-4.8835	0.7679	0.0000	C	1.1414	4.0381	0.0000	C	-2.1943	-3.7341	0.0000
C	-2.7161	3.0687	0.0000	C	-3.7479	-1.4385	0.0000	C	2.8119	0.1133	0.0000	C	0.2499	-4.0098	0.0000
C	-2.0266	4.2976	0.0000	C	-3.8605	-2.8444	0.0000	C	4.5593	2.5212	0.0000	C	1.3622	-4.8882	0.0000
C	-4.1393	3.1027	0.0000	C	-4.9493	-0.6732	0.0000	C	2.5611	3.9127	0.0000	C	-1.0530	-4.5914	0.0000
C	-2.7029	5.5093	0.0000	C	-5.0964	-3.4725	0.0000	C	3.3950	5.0578	0.0000	C	1.1973	-6.2597	0.0000
C	-4.8082	4.3428	0.0000	C	-6.1967	-1.3314	0.0000	C	5.3509	3.6521	0.0000	C	-1.1973	-5.9995	0.0000
C	-4.1047	5.5363	0.0000	C	-6.2766	-2.7131	0.0000	C	4.7675	4.9360	0.0000	C	-0.0933	-6.8256	0.0000
H	1.2693	-4.3502	0.0000	H	2.9676	3.4593	0.0000	H	-4.0735	0.5191	0.0000	H	0.2144	7.9047	0.0000
H	3.3218	-5.7276	0.0000	H	7.1027	0.7309	0.0000	H	-5.5374	-1.4572	0.0000	H	2.1998	6.4194	0.0000
H	-0.7824	-2.9753	0.0000	H	5.8180	-1.3225	0.0000	H	-1.8944	1.4859	0.0000	H	-2.0702	6.9058	0.0000
H	6.1018	-1.5789	0.0000	H	1.2014	2.4904	0.0000	H	-5.8448	-3.8655	0.0000	H	-2.3699	4.4898	0.0000
H	4.0475	-0.2045	0.0000	H	5.1481	4.5572	0.0000	H	0.2571	-2.9697	0.0000	H	3.1826	4.1863	0.0000
H	1.9991	1.1697	0.0000	H	3.6641	-2.5076	0.0000	H	-0.9428	-4.6009	0.0000	H	2.9629	1.7745	0.0000
H	5.7836	-5.8725	0.0000	H	1.2463	-2.4787	0.0000	H	-4.9305	-6.1692	0.0000	H	-2.5538	2.5218	0.0000
H	-2.8326	-1.5807	0.0000	H	7.2440	3.2061	0.0000	H	-0.7394	3.0821	0.0000	H	-2.7773	0.1455	0.0000
H	7.1720	-3.8007	0.0000	H	-1.2463	2.4787	0.0000	H	-2.4610	-6.5141	0.0000	H	2.7773	-0.1455	0.0000
H	-0.0205	2.5229	0.0000	H	-1.2014	-2.4904	0.0000	H	2.4453	-1.9809	0.0000	H	2.5538	-2.5218	0.0000
H	-4.8527	-0.2554	0.0000	H	-3.6641	2.5076	0.0000	H	0.7068	5.0342	0.0000	H	-2.9629	-1.7745	0.0000
H	-5.9748	1.9300	0.0000	H	-5.8180	1.3225	0.0000	H	3.8854	-0.0303	0.0000	H	-3.1826	-4.1863	0.0000
H	-0.9425	4.3106	0.0000	H	-2.9676	-3.4593	0.0000	H	5.0451	1.5528	0.0000	H	2.3699	-4.4898	0.0000
H	-5.8952	4.3490	0.0000	H	-7.1027	-0.7309	0.0000	H	2.9286	6.0396	0.0000	H	2.0702	-6.9058	0.0000
H	-2.1407	6.4383	0.0000	H	-5.1481	-4.5572	0.0000	H	6.4322	3.5508	0.0000	H	-2.1998	-6.4194	0.0000
H	-4.6341	6.4843	0.0000	H	-7.2440	-3.2061	0.0000	H	5.3978	5.8202	0.0000	H	-0.2144	-7.9047	0.0000
		0201				0220				1001				1002	
	x	y	z		x	y	z		x	y	z		x	y	z
C	-2.6965	3.0397	0.0000	C	0.0000	0.0000	0.7191	C	0.0000	0.0000	1.4557	C	3.7845	1.1956	0.2904
C	-1.9657	4.2372	0.0000	C	0.0000	0.0000	2.1394	C	-0.0279	1.2909	0.7740	C	4.1518	-0.1420	0.0757

C	-2.0689	1.7943	0.0000	C	-0.1247	1.2778	0.0431	C	0.0000	0.0000	2.8654	C	2.4554	1.6146	0.2477
C	-2.5885	5.5207	0.0000	C	0.1247	-1.2778	0.0431	C	0.0279	-1.2909	0.7740	C	5.5058	-0.5876	0.1088
C	-0.5289	4.1615	0.0000	C	-0.5703	1.3836	-1.3178	C	-0.4115	1.4625	-0.5616	C	3.1163	-1.0859	-0.2433
C	-0.6301	1.7084	0.0000	C	0.0773	2.4855	0.7688	C	0.2594	2.4902	1.5354	C	1.3948	0.6487	0.0500
C	-2.8313	0.5800	0.0000	C	0.1224	1.2281	2.8467	C	-0.2257	-1.2150	3.5949	C	2.1337	3.0136	0.3032
C	0.2202	5.3759	0.0000	C	0.5703	-1.3836	-1.3178	C	0.2257	1.2150	3.5949	C	3.4836	-2.4319	-0.5391
C	-1.8343	6.6666	0.0000	C	-0.1224	-1.2281	2.8467	C	-0.2594	-2.4902	1.5354	C	5.8202	-1.8946	-0.1679
C	0.0944	2.9011	0.0000	C	-0.0773	-2.4855	0.7688	C	0.4115	-1.4625	-0.5616	C	1.7748	-0.6611	-0.2552
C	-2.2304	-0.6377	0.0000	C	0.0627	3.7612	0.0731	C	0.2998	3.7322	0.8988	C	0.0046	1.1113	0.0706
C	0.0000	0.3891	0.0000	C	-0.6626	2.5842	-1.9572	C	-0.4041	2.7143	-1.1994	C	0.8626	3.4373	0.0953
C	-0.4127	6.5932	0.0000	C	0.2244	2.4182	2.1851	C	0.4172	2.3987	2.9564	C	4.7975	-2.8270	-0.4991
C	-0.7965	-0.7830	0.0000	C	-0.0627	-3.7612	0.0731	C	0.4041	-2.7143	-1.1994	C	-1.1427	0.2430	0.1950
C	1.4106	0.2399	0.0000	C	0.6626	-2.5842	-1.9572	C	-0.4172	-2.3987	2.9564	C	-0.2241	2.5046	-0.0420
C	-0.1700	-2.0842	0.0000	C	-0.2244	-2.4182	2.1851	C	-0.2998	-3.7322	0.8988	C	-1.0450	-1.0900	0.7247
C	2.0080	-0.9933	0.0000	C	-0.3012	3.8012	-1.3060	C	-0.7869	2.8666	-2.5661	C	-1.5392	2.9864	-0.2599
C	-0.9428	-3.2935	0.0000	C	0.3986	4.9869	0.7009	C	0.0000	3.8814	-0.4621	C	-2.4462	0.7409	-0.1046
C	1.2485	-2.1918	0.0000	C	-0.3986	-4.9869	0.7009	C	0.0000	-3.8814	-0.4621	C	-3.5878	-0.1615	-0.0970
C	1.8829	-3.5006	0.0000	C	0.3012	-3.8012	-1.3060	C	0.7869	-2.8666	-2.5661	C	-2.1255	-1.9146	0.8113
C	-0.3551	-4.5219	0.0000	C	-0.3310	5.0389	-1.9922	C	0.0284	5.1432	-1.1278	C	-2.6040	2.1298	-0.3524
C	3.2882	-3.6806	0.0000	C	0.3729	6.1818	0.0083	C	-0.0284	-5.1432	-1.1278	C	-4.8810	0.2275	-0.5248
C	1.0660	-4.6694	0.0000	C	0.3310	-5.0389	-1.9922	C	0.7492	-4.0957	-3.1738	C	-3.4152	-1.5026	0.3555
C	1.6667	-5.9505	0.0000	C	-0.3729	-6.1818	0.0083	C	-0.7492	4.0957	-3.1738	C	-4.5192	-2.3868	0.3792
C	3.8536	-4.9415	0.0000	C	0.0000	6.2137	-1.3505	C	0.3329	-5.2475	-2.4468	C	-5.9436	-0.6561	-0.5026
C	3.0389	-6.0908	0.0000	C	0.0000	-6.2137	-1.3505	C	-0.3329	5.2475	-2.4468	C	-5.7669	-1.9755	-0.0424
H	-3.7833	3.0825	0.0000	H	-0.9040	0.4917	-1.8310	H	-0.7748	0.6166	-1.1303	H	4.5634	1.9368	0.4539
H	-3.6741	5.5724	0.0000	H	0.1524	1.2004	3.9326	H	0.7748	-0.6166	-1.1303	H	6.2850	0.1295	0.3535
H	-3.9156	0.6531	0.0000	H	0.9040	-0.4917	-1.8310	H	0.2732	1.1594	4.6791	H	2.9414	3.7252	0.4514
H	1.3057	5.3184	0.0000	H	-0.1524	-1.2004	3.9326	H	-0.2732	-1.1594	4.6791	H	2.7003	-3.1429	-0.7887
H	-2.3188	7.6385	0.0000	H	-1.0316	2.6292	-2.9784	H	0.5325	4.6159	1.4886	H	6.8546	-2.2242	-0.1397
H	1.1788	2.8858	0.0000	H	0.3453	3.3285	2.7595	H	-0.5325	-4.6159	1.4886	H	1.0266	-1.3844	-0.5538
H	-2.8585	-1.5192	0.0000	H	-0.3453	-3.3285	2.7595	H	0.6345	3.3049	3.5150	H	0.6311	4.4985	0.0630
H	0.1693	7.5101	0.0000	H	1.0316	-2.6292	-2.9784	H	-0.6345	-3.3049	3.5150	H	5.0642	-3.8563	-0.7199
H	2.0466	1.1169	0.0000	H	0.7068	4.9979	1.7399	H	1.1012	-1.9856	-3.1198	H	-0.0991	-1.4232	1.1296
H	3.0903	-1.0387	0.0000	H	-0.7068	-4.9979	1.7399	H	-1.1012	1.9856	-3.1198	H	-1.6895	4.0568	-0.3708
H	-2.0240	-3.2422	0.0000	H	-0.6178	5.0453	-3.0405	H	1.0366	-4.1977	-4.2161	H	-2.0201	-2.9039	1.2487
H	-0.9689	-5.4188	0.0000	H	0.6460	7.1026	0.5152	H	-0.3384	-6.0211	-0.5671	H	-3.5879	2.5371	-0.5514
H	3.9469	-2.8201	0.0000	H	0.6178	-5.0453	-3.0405	H	0.3384	6.0211	-0.5671	H	-5.0496	1.2298	-0.9013
H	1.0234	-6.8266	0.0000	H	-0.6460	-7.1026	0.5152	H	-1.0366	4.1977	-4.2161	H	-4.3647	-3.4023	0.7346
H	4.9346	-5.0455	0.0000	H	-0.0215	7.1575	-1.8870	H	0.3077	-6.2123	-2.9447	H	-6.9206	-0.3299	-0.8467
H	3.4902	-7.0784	0.0000	H	0.0215	-7.1575	-1.8870	H	-0.3077	6.2123	-2.9447	H	-6.6070	-2.6632	-0.0243
		1012				1021				1101				1102	
	x	y	z		x	y	z		x	y	z		x	y	z
C	0.4270	4.1473	0.0000	C	-0.0013	0.7049	0.0000	C	-2.5465	-0.4829	0.0000	C	-2.1200	-0.8557	0.0000
C	1.8239	4.3100	0.0000	C	1.2598	1.4372	0.0000	C	-2.4537	0.9304	0.0000	C	-1.4335	-2.0889	0.0000
C	-0.1692	2.8898	0.0000	C	-1.2598	1.3969	0.0000	C	-1.4261	-1.2949	0.0000	C	-1.4497	0.3596	0.0000
C	2.4494	5.5900	0.0000	C	0.0013	-0.7049	0.0000	C	-3.5821	1.7650	0.0000	C	-2.0973	-3.3292	0.0000

C	2.6488	3.1336	0.0000	C	1.3359	2.8331	0.0000	C	-1.1364	1.5254	0.0000	C	0.0136	-2.0635	0.0000
C	-1.6058	2.7561	0.0000	C	2.4989	0.6973	0.0000	C	-0.1001	-0.7040	0.0000	C	0.0000	0.3958	0.0000
C	0.6537	1.7047	0.0000	C	-2.4466	0.7326	0.0000	C	-1.5605	-2.7339	0.0000	C	-2.1687	1.6014	0.0000
C	4.0650	3.2899	0.0000	C	1.2598	-1.3969	0.0000	C	-1.0210	2.9242	0.0000	C	0.7149	-3.2831	0.0000
C	2.0353	1.8650	0.0000	C	-1.2598	-1.4372	0.0000	C	-3.4645	3.1575	0.0000	C	-1.3966	-4.5350	0.0000
C	-2.2035	1.5409	0.0000	C	3.7200	1.3741	0.0000	C	0.0000	0.6748	0.0000	C	0.6799	-0.8152	0.0000
C	0.0000	0.3905	0.0000	C	2.5602	3.5211	0.0000	C	1.0736	-1.5946	0.0000	C	0.6753	1.6955	0.0000
C	3.8182	5.7027	0.0000	C	2.4466	-0.7326	0.0000	C	-0.4807	-3.5477	0.0000	C	-1.5232	2.7948	0.0000
C	-1.4317	0.3219	0.0000	C	-2.4989	-0.6973	0.0000	C	-4.6058	4.0206	0.0000	C	-2.0595	-5.8048	0.0000
C	0.7021	-0.8141	0.0000	C	-1.3359	-2.8331	0.0000	C	-2.1453	3.7547	0.0000	C	0.0534	-4.5111	0.0000
C	4.6352	4.5395	0.0000	C	2.6266	4.9473	0.0000	C	0.8667	-3.0222	0.0000	C	-0.0841	2.8868	0.0000
C	0.0625	-2.0635	0.0000	C	3.7907	2.7736	0.0000	C	2.3798	-1.1234	0.0000	C	2.0994	1.7878	0.0000
C	-2.0711	-0.9209	0.0000	C	-2.5602	-3.5211	0.0000	C	-2.0402	5.1815	0.0000	C	0.7578	-5.7582	0.0000
C	0.8306	-3.2776	0.0000	C	-3.7200	-1.3741	0.0000	C	3.4976	-1.9854	0.0000	C	2.7421	2.9932	0.0000
C	-1.3678	-2.1321	0.0000	C	5.0289	3.4836	0.0000	C	1.9588	-3.8810	0.0000	C	0.5845	4.1714	0.0000
C	0.2247	-4.4931	0.0000	C	-3.7907	-2.7736	0.0000	C	-4.4597	5.3817	0.0000	C	-1.3459	-6.9716	0.0000
C	-2.0173	-3.4391	0.0000	C	-2.6266	-4.9473	0.0000	C	4.8330	-1.4929	0.0000	C	-0.1091	5.4108	0.0000
C	-3.4208	-3.6016	0.0000	C	3.8350	5.5956	0.0000	C	3.2846	-3.4047	0.0000	C	2.0138	4.2157	0.0000
C	-1.2080	-4.6135	0.0000	C	-5.0289	-3.4836	0.0000	C	-3.1590	5.9703	0.0000	C	0.0833	-6.9481	0.0000
C	-4.0021	-4.8577	0.0000	C	-3.8350	-5.5956	0.0000	C	5.9022	-2.3571	0.0000	C	0.5654	6.6144	0.0000
C	-1.8234	-5.8852	0.0000	C	5.0514	4.8546	0.0000	C	4.4136	-4.2711	0.0000	C	2.6824	5.4652	0.0000
C	-3.1994	-6.0121	0.0000	C	-5.0514	-4.8546	0.0000	C	5.6902	-3.7612	0.0000	C	1.9760	6.6474	0.0000
H	-0.2060	5.0318	0.0000	H	-1.2734	2.4797	0.0000	H	-3.5328	-0.9410	0.0000	H	-3.2075	-0.8601	0.0000
H	1.8214	6.4771	0.0000	H	0.4329	3.4331	0.0000	H	-4.5723	1.3151	0.0000	H	-3.1847	-3.3450	0.0000
H	-2.2026	3.6643	0.0000	H	1.2734	-2.4797	0.0000	H	-2.5643	-3.1502	0.0000	H	-3.2549	1.5666	0.0000
H	4.6881	2.3993	0.0000	H	-3.3827	1.2844	0.0000	H	-0.0304	3.3733	0.0000	H	1.8025	-3.2663	0.0000
H	2.6867	0.9972	0.0000	H	4.6418	0.7969	0.0000	H	0.9731	1.1548	0.0000	H	1.7638	-0.8399	0.0000
H	-3.2873	1.4600	0.0000	H	-0.4329	-3.4331	0.0000	H	-0.6047	-4.6273	0.0000	H	-2.1139	3.7023	0.0000
H	4.2845	6.6835	0.0000	H	3.3827	-1.2844	0.0000	H	2.5769	-0.0563	0.0000	H	2.6961	0.8839	0.0000
H	1.7870	-0.8127	0.0000	H	-4.6418	-0.7969	0.0000	H	-5.5951	3.5705	0.0000	H	-3.1462	-5.8205	0.0000
H	5.7160	4.6453	0.0000	H	1.6975	5.5116	0.0000	H	1.7863	-4.9549	0.0000	H	3.8280	3.0328	0.0000
H	-3.1560	-0.9225	0.0000	H	-1.6975	-5.5116	0.0000	H	-1.0491	5.6276	0.0000	H	1.8445	-5.7381	0.0000
H	1.9146	-3.2014	0.0000	H	5.9547	2.9143	0.0000	H	-5.3351	6.0244	0.0000	H	-1.8620	-7.9271	0.0000
H	0.8170	-5.4042	0.0000	H	3.8715	6.6809	0.0000	H	4.9934	-0.4178	0.0000	H	-1.1922	5.4281	0.0000
H	-4.0662	-2.7305	0.0000	H	-3.8715	-6.6809	0.0000	H	-3.0640	7.0521	0.0000	H	0.6306	-7.8860	0.0000
H	-1.1913	-6.7695	0.0000	H	-5.9547	-2.9143	0.0000	H	4.2474	-5.3452	0.0000	H	3.7693	5.4719	0.0000
H	-5.0841	-4.9499	0.0000	H	5.9992	5.3848	0.0000	H	6.9163	-1.9689	0.0000	H	0.0045	7.5443	0.0000
H	-3.6591	-6.9959	0.0000	H	-5.9992	-5.3848	0.0000	H	6.5446	-4.4315	0.0000	H	2.4976	7.5998	0.0000

	1111		
	x	y	z
C	0.0000	0.0000	0.7306
C	0.0000	0.0000	-0.7306
C	0.0000	1.2317	1.4102
C	0.0000	-1.2317	1.4102
C	0.0000	2.4532	0.7300
C	0.0000	1.2317	-1.4102

C 0.0000 -2.4532 0.7300<br>C 0.0000 -1.2317 -1.4102<br>C 0.0000 3.6967 1.4092<br>C 0.0000 2.4532 -0.7300<br>C 0.0000 -3.6967 1.4092<br>C 0.0000 -2.4532 -0.7300<br>C 0.0000 4.9049 0.7287<br>C 0.0000 3.6967 -1.4092<br>C 0.0000 -4.9049 0.7287<br>C 0.0000 -3.6967 -1.4092<br>C 0.0000 6.1690 1.4113<br>C 0.0000 4.9049 -0.7287<br>C 0.0000 -6.1690 1.4113<br>C 0.0000 -4.9049 -0.7287<br>C 0.0000 6.1690 -1.4113<br>C 0.0000 7.3439 0.7172<br>C 0.0000 -7.3439 0.7172<br>C 0.0000 -6.1690 -1.4113<br>C 0.0000 7.3439 -0.7172<br>C 0.0000 -7.3439 -0.7172<br>H 0.0000 1.2319 2.4976<br>H 0.0000 -1.2319 2.4976<br>H 0.0000 1.2319 -2.4976<br>H 0.0000 -1.2319 -2.4976<br>H 0.0000 3.6969 2.4967<br>H 0.0000 -3.6969 2.4967<br>H 0.0000 3.6969 -2.4967<br>H 0.0000 -3.6969 -2.4967<br>H 0.0000 6.1672 2.4981<br>H 0.0000 -6.1672 2.4981<br>H 0.0000 6.1672 -2.4981<br>H 0.0000 8.2917 1.2472<br>H 0.0000 -6.1672 -2.4981<br>H 0.0000 -8.2917 1.2472<br>H 0.0000 8.2917 -1.2472<br>H 0.0000 -8.2917 -1.2472			
